# Advances in orofacial pain research: a bibliometric analysis

**DOI:** 10.3389/fneur.2025.1609437

**Published:** 2025-08-14

**Authors:** Shuo Qin, Juan Liu, JiaXing Fan, Zhe Qin, Jing Jia

**Affiliations:** ^1^Department of Stomatology, Third Medical Center of PLA General Hospital, Beijing, China; ^2^Third Clinical School of Medicine, Jinzhou Medical University, Jinzhou, China; ^3^Department of Stomatology, Zaozhuang Hospital, Shandong Health Group, Zaozhuang, China

**Keywords:** orofacial pain, bibliometric analysis, cooperative network analysis, co-occurrence network analysis, keyword analysis

## Abstract

**Objective:**

Orofacial pain has become increasingly prevalent with the advancement of society and economy. Bibliometrics, an interdisciplinary field encompassing mathematics, statistics, and information science, offers insights into the trends, research focal points, and knowledge framework of orofacial pain through quantitative analysis of relevant literature. This study aims to systematically map the evolutionary trajectory of orofacial pain research from 2000 to 2024. It will analyze publication trends, collaborative networks, and emerging hotspots to provide data-driven guidance for future research directions and resource allocation.

**Methods:**

This study employed bibliometric analysis to examine literature published between 2000 and 2024 using keywords such as “face pain,” “craniofacial pain,” “neuralgic facial pain,” “myofacial pain,” “oral-maxillofacial pain,” “oral and maxillofacial pain,” and “orofacial pain.” Utilizing tools like CiteSpace and VOSviewer, we conducted trend analysis on publication volume, constructed author collaboration networks, and performed keyword co-occurrence analysis.

**Results:**

Our analysis revealed a rising publication trend in the field, the establishment of a core group of authors, continuous expansion of collaboration networks, and current research focal points on “diagnostic criteria,” “manual therapy,” “systematic review,” “quality,” “joint disorders,” “scale,” and “care.”

**Conclusion:**

This study demonstrates that bibliometrics offers a comprehensive and objective quantitative analysis for academic research, aiding researchers in understanding disciplinary developments, providing a scientific foundation for future research directions and resource allocation, and fostering sustainable disciplinary growth and innovation.

## 1 Introduction

Oral and maxillofacial pain encompasses a spectrum of discomfort in the facial and oral regions, ranging from mild to severe, impacting both the functionality of these areas and the overall well-being of individuals. This pain can arise from various sources, including local structural diseases or functional disorders within the oral cavity and jaw, such as dental caries-induced pulpitis, periodontitis-related gum pain, and temporomandibular joint disorders. Additionally, neurological issues like trigeminal neuralgia and glossopharyngeal neuralgia, as well as tumor metastasis to the oral and maxillofacial regions, can also contribute to this type of pain. The diagnosis of oral and maxillofacial pain is multifaceted, with diverse criteria (1). However, the 2020 International Classification of Orofacial Pain (ICOP), developed collaboratively by leading international professional organizations in the field, comprehensively delineates various types of oral and maxillofacial pain and offers precise diagnostic guidelines, serving as a crucial resource for clinicians (2).

Research on the etiology and treatment of oral and maxillofacial pain is a prominent area of interest among scholars (3). A landmark initiative in this field is the Orofacial Pain: Prospective Evaluation and Risk Assessment (OPERA) project, which was launched in 2006 to identify risk factors for painful temporomandibular disorders (TMD). Over the course of a decade, the project recruited 3,258 TMD-free adults across four U.S. sites, assessing various factors including genetic, biological, psychosocial, clinical, and health status. Key findings revealed that the development of TMD is driven by a biopsychosocial interplay: genetic predispositions (e.g., variants in pain-related genes), phenotypic traits (e.g., heightened sensitivity to experimental pain), psychosocial stressors (e.g., anxiety, somatization), and clinical factors (e.g., prior jaw injuries) collectively contribute to the risk (4). This underscores the biopsychosocial nature of orofacial pain, where biological vulnerabilities, psychological states, and social contexts dynamically interact to modulate pain perception and progression.

Studies have delved into various aspects such as the impact of estrogen on oral and maxillofacial pain (5–7), the regulatory role of the trigeminal nerve (8–10), sympathetic-parasympathetic interactions in temporomandibular arthritis (11), involvement of prefrontal cortex neurons in chronic pain (12–14), neurovascularization, signal pathways, ion channels, and receptors (15–17). Central sensitization, a key mechanism underlying chronic orofacial pain, has garnered significant attention in recent years. Studies indicate that prolonged nociceptive input induces plastic changes in the central nervous system, which in turn heightens the responsiveness of spinal and supraspinal neurons to mild stimuli. This phenomenon is associated with an increased expression of pro-inflammatory cytokines, such as IL-6 and TNF-α, as well as alterations in glutamate signaling within the trigeminal system, thereby amplifying pain persistence (18, 19).

Additionally, investigations (20–22) have explored the interplay between nerves and psychology (23–25), central sensitization, emotional regulation (26–28), and various treatment modalities including neuromodulation techniques like optogenetics and chemogenetics, molecular targeted therapies, and drugs promoting neuronal autophagy (29–31). Psycho-emotional factors play a critical role in the perception of orofacial pain (OFP) and headaches (HA). Anxiety, stress, and depression are correlated with worsened sleep quality, insomnia, and daytime sleepiness, which exacerbate pain and diminish treatment responses. Therefore, systematic evaluation of psychosocial factors is therefore critical (32).

Multifactorial analyses indicate that the pain and headaches experienced by patients with temporomandibular disorder (TMD) are significantly associated with sleep bruxism (SB) and comorbidities such as a history of cancer and gastroesophageal reflux disease (GERD). This underscores the necessity of addressing both systemic and lifestyle factors (33). These therapeutic approaches are often combined with physiotherapy (34–36), psychotherapy, and traditional Chinese medicine to modulate neurotransmitters and provide comprehensive care for patients with oral and maxillofacial pain (37, 38). Emerging treatments include botulinum toxin, which demonstrates promise in alleviating muscle hyperactivity and pain in temporomandibular disorders (TMD) and trigeminal neuralgia through its neuromodulatory effects (39). Additionally, injectable platelet-rich fibrin (I-PRF) has been shown to provide significant pain relief in temporomandibular joint (TMJ) disorders following single articular cavity injections (40).

In the field of orofacial pain research, existing results have thoroughly explored core issues related to the comprehensive mechanisms of orofacial pain, including trigeminal nerve biology and inflammatory pain pathways. Concurrently, clinical management strategies from previous studies have enhanced our understanding of orofacial pain. However, there is a notable lack of systematic descriptions of the scientific evolution in this field. This study addresses this gap by providing a systematic, data-driven overview of the evolutionary trajectories in orofacial pain research. This critical gap hinders researchers from identifying hidden trends and cutting-edge opportunities, thereby limiting their collaborative efforts and ability to explore unknown territories. To address this, we transcend the limitations of traditional reviews and, for the first time, employ bibliometrics (using the Citespace and VOSviewer tools) to construct a multidimensional “panorama map” for the years 2000 to 2024. This map illustrates an academic star chart of author collaborations, institutional contributions, and interdisciplinary intersections. It reveals emerging hotspots, such as the significant rise of “quantitative sensory testing” and “systematic reviews,” while also emphasizing their importance in understanding the relationship between human and animal physiology. Furthermore, it highlights the active and dormant research clusters in the US, Pakistan, and Africa. The value of this multidimensional analysis lies in its actionable insights, which provide a strategic roadmap for researchers. First, it aids in identifying subfields, such as temporomandibular disorders and trigeminal neuralgia, that are over-researched, while emphasizing the need for greater focus on the mechanisms of idiopathic orofacial pain. Second, it reveals cooperation gaps, such as the limited inter-agency partnerships in China, which, if addressed, could accelerate innovation. Finally, by linking keyword co-occurrence data, such as “manual treatment” and “quality of life,” with clinical needs, we establish a strategic bridge between basic research and patient requirements, guiding resource allocation toward patient-centered priorities. Ultimately, our bibliometric study transcends mere descriptive summaries, offering actionable insights that propel the development of targeted and impactful research in the field of orofacial pain.

To address the fragmentation of oral and facial pain research, which is often simply categorized as “general pain” or “dental complications,” this study conducts a systematic bibliometric analysis of 3,372 publications from 2000 to 2024. It focuses on three core issues: (1) quantifying long-term output trends in the field and identifying inflection points driven by key events such as updates to diagnostic standards or technological advances; (2) revealing the differences and collaboration patterns among leading countries, institutions, and research networks in terms of quality (citations, methodology) and focus (basic mechanisms vs. clinical applications); and (3) identifying how emerging hotspots, such as central sensitization, psycho-emotional regulation, and novel interventions, map onto unmet clinical needs or scientific breakthroughs. By addressing these questions, this study aims to provide a comprehensive, data-driven overview of orofacial pain research and offer insights for future studies. It establishes an operational framework for prioritizing future research agendas, enhancing international collaboration, and accurately aligning with clinical needs. Utilizing mathematical and statistical methods, bibliometrics evaluates and predicts the developmental status, growth trends, and evolutionary trajectories of scientific and technological fields within the framework of literature systems. This study employs bibliometric analysis to focus specifically on the domain of oral and maxillofacial pain, systematically organizing national scientific research trends, institutional academic performance, CORE journal distribution, author contributions, and key research keywords. The objective of this study is to quantitatively assess the academic achievements of researchers and the scientific research capabilities of higher education institutions, thereby providing practical guidance for scientific research practitioners. The primary aim of this bibliometric analysis is to systematically present the overall pattern of global research on orofacial pain from 2000 to 2024. This includes an analysis of publication trends, collaboration networks among countries, institutions, and authors, as well as an examination of the evolution of research on oral and maxillofacial pain over time. Additionally, it identifies key research topics and emerging frontiers, ultimately offering data support for future research priorities, the promotion of international cooperation, and the optimization of resource allocation in this field.

## 2 Methods

### 2.1 Data sources and searches

We utilized SCI-EXPANDED, a high-quality digital bibliographic resource database from Clarivate Analytics’ Web of Science Core Database, as our primary research source. This database is widely recognized by researchers as the most suitable option for bibliometric analysis. To enhance the comprehensiveness of our search scope, we noted that while SCOPUS offers broader journal coverage, a previous study indicated a 92% overlap with WOS in the literature concerning orofacial pain. Consequently, SCOPUS did not significantly differ from WOS, with few unique records contributing new insights. Additionally, PubMed lacks comprehensive citation data and essential author/institutional metadata for collaboration networks and citation analysis, rendering it less suitable for our multidimensional bibliometric framework. Resource constraints limited our ability to conduct parallel analyses across multiple databases; therefore, we summarized and supplemented keywords based on MeSH terms and CNKI. Our search query was constructed as follows: ts = (“facial pain” or “craniofacial pain” or “neurofacial pain” or “musculofacial pain” or “oral-maxillofacial pain” or “oral and maxillofacial pain” or “orofacial pain”), targeting articles published between January 1, 2000, and December 31, 2024. Recognizing that English is the dominant language of scholarly publications and that the major categories of articles are already citation-dominated, we restricted our search to articles and reviews published in English. This study adhered to the principles outlined in the Declaration of Helsinki. We identified duplicates using title and DOI matching through R’s bibliometric package, ensuring the removal of duplicates to maintain unique records. For quality screening, we did not employ formal quality scores such as the CASP checklist, as bibliometric analyses often prioritize coverage breadth over the rigor of individual studies. However, we excluded multiple articles with fewer than five citations to mitigate noise from low-impact studies.

### 2.2 Data analysis and visualization

**Figure 1:**
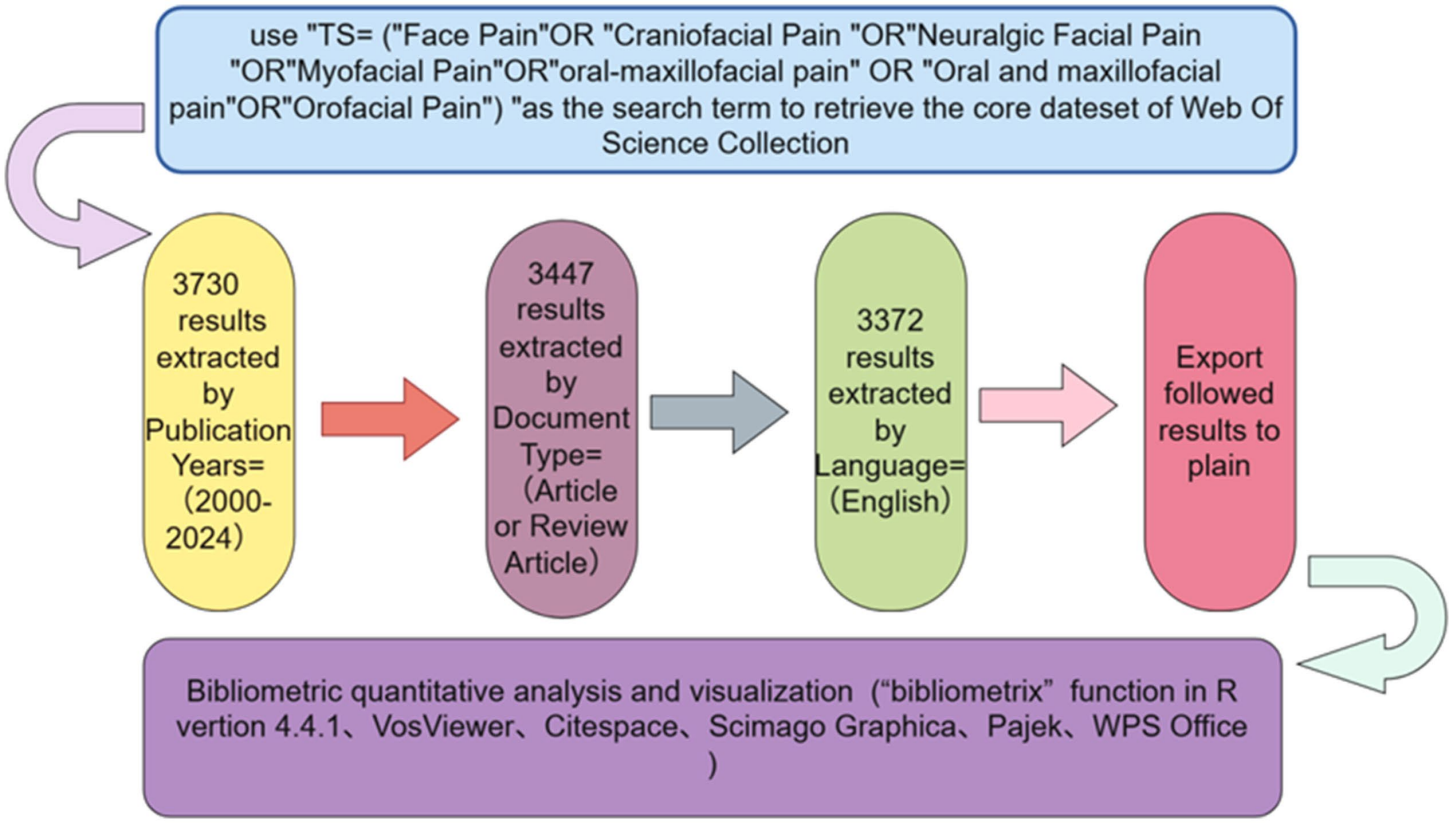
Figdraw flow chart. The flowchart illustrates the systematic search and screening process employed in this study. The blue boxes represent the initial records identified from the Web of Science Core Collection, while the grey boxes denote the successive exclusion steps, which include the removal of duplicates, non-English publications, and non-article/review materials. Ultimately, the green box indicates the final count of publications included in the bibliometric analysis, totaling 3,372.

Two independent researchers conducted the study to ensure result reliability. Literature was retrieved in “plain text” format, and relevant information was extracted for analysis. VOSviewer and Scimago Graphic were utilized for author, country/region, and institution visualization and quantification. CiteSpace6.3R2 was employed for keyword clustering, emergent word analysis, and reference data visualization. Data extraction and compilation were done using R language, with Pajek used for auxiliary graphics conversion. WPS Office was utilized to analyze and graphically represent the number of articles published per country/region and trends in publication numbers (see Figure 1).

## 3 Results

### 3.1 Annual publications and trends

**Figure 2:**
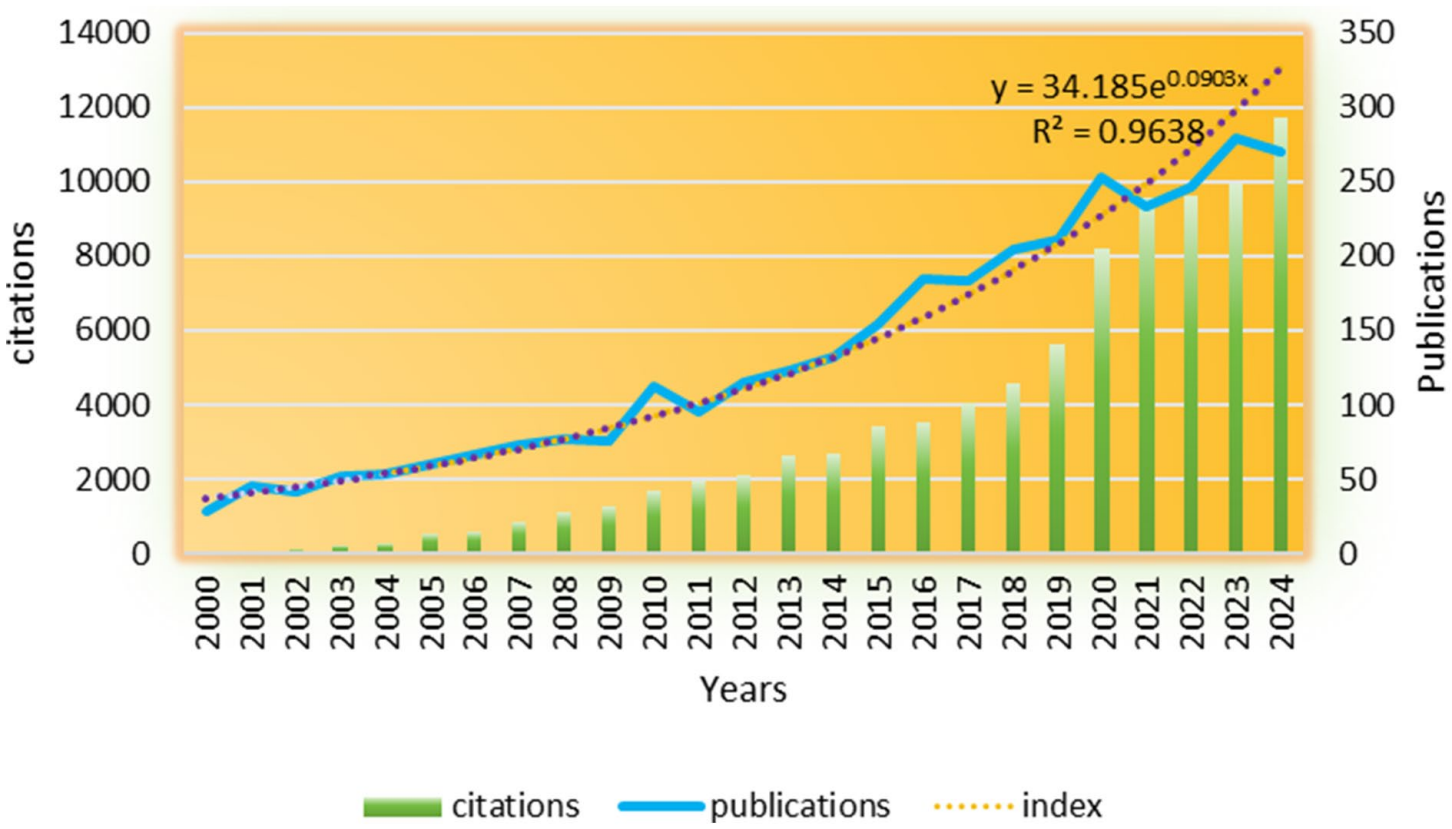
The annual publication output and citation trend from 2000 to 2024 is illustrated in the figure. The bars, representing the number of articles published each year (left *y*-axis), are complemented by the red line, which indicates the cumulative citations received by publications from each corresponding year (right *y*-axis). An exponential curve, described by the equation *y* = 34.185e^(0.0903*x*)^ with an *R*^2^ value of 0.9638, has been superimposed to emphasize the significant growth observed following the inflection points in 2009 and 2019.

2,751 studies (81.7%) and 533 reviews (15.17%) were included, encompassing 86 countries/regions and 2,919 institutions. Research on oral and maxillofacial pain has shown a notable increase from 2000 to 2024, as evidenced by the growing body of literature. Figure 2 illustrates the annual publication volume during this period, segmented into four stages: a slow growth period (2000–2009), a first peak period (2009–2011), a second peak period (2019–2021), and a rapid growth period (2011–2024). Prior to 2009, the publication rate exhibited gradual growth, surpassing 28 articles. Subsequent to 2009, there was a significant acceleration in publications, with over 96 articles being published annually, culminating in a peak of 280 articles in 2023. The citations of these related works increased steadily each year, reaching a pinnacle of 11,730 in 2024. Publications showed exponential growth (*y* = 34.185e0.0903*x*, *R*^2^ = 0.9638), with two critical inflection points: 2009 (first peak) and 2019 (second peak). The 2009 surge coincided with the publication of the first International RDC/TMD diagnostic criteria (41), standardizing research endpoints and facilitating cross-study comparisons. The 2019 peak aligned with increased funding for chronic pain research, most notably the US National Institutes of Health’s Helping to End Addiction Long-term (HEAL) Initiative (NIH Guide NOT-NS-19-024, 4 April 2019), and the adoption of telemedicine during the COVID-19 pandemic. Citation growth (peaking at 11,730 in 2024) outpaced publication volume, suggesting improving research influence, though 15% of articles received <5 citations, indicating potential variability in quality.

### 3.2 Number of publications by country

**Figure 3:**
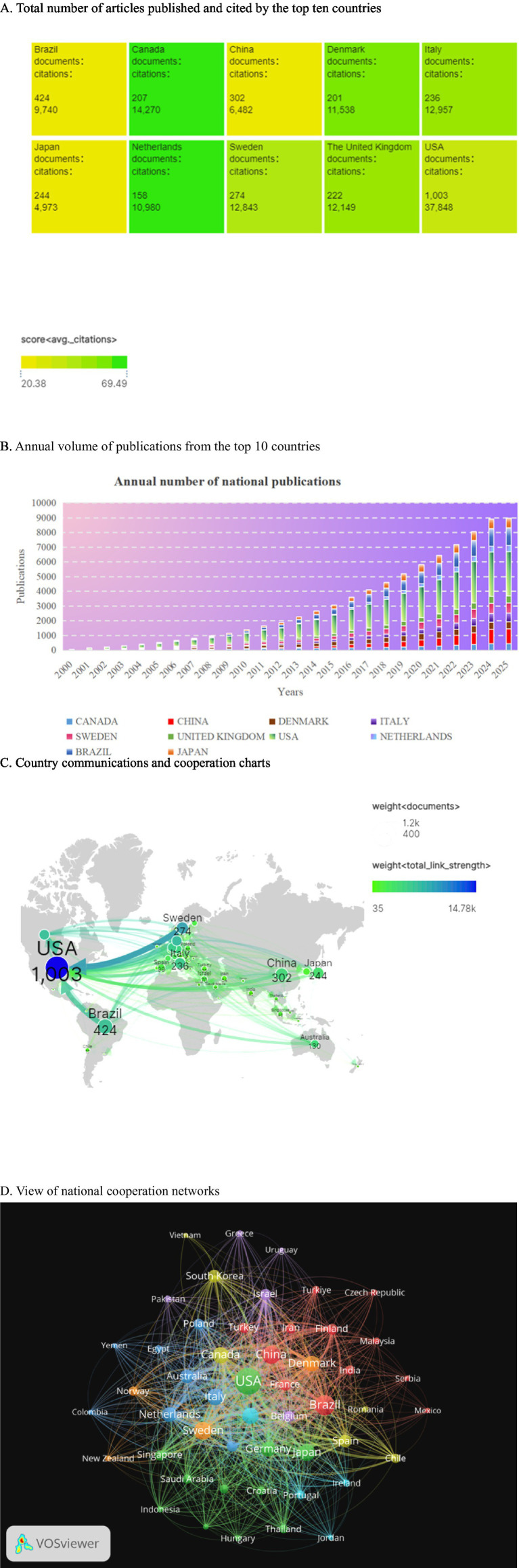
This section analyzes global contribution and collaboration patterns in academic publishing. It includes the following components: **(A)** A summary of total articles and citations for the 10 most productive countries. **(B)** Yearly publication counts for these countries. **(C)** A chord diagram illustrating the number of co-authored papers between country pairs, with arc width representing collaboration intensity. **(D)** A network map generated using CiteSpace, where each node represents a country. The size of each node corresponds to the total number of publications, and the thickness of the connecting lines indicates the level of co-authorship. Notably, the United States serves as the central hub in this network, while European Union countries, Brazil, and China form dense peripheral clusters, highlighting significant patterns of international collaboration.

A total of 86 countries or territories conducted and published studies, and the top 10 countries by number of publications are shown in Figure 3A. The United States led with 1,003 papers, followed by Brazil (424), China (302), Sweden (274) and Japan (244). Italy, the UK, Canada, Denmark and the Netherlands each received more than 10,000 citations, although they did not make the top five. Figure 3B shows the upward trend in the number of annual publications in the top 10 countries, especially in the United States, China and the Netherlands. Figure 3C highlights frequent exchanges and collaborations mainly involving Denmark, China, Brazil and Japan, with the United States as the central hub. Figure 3D visually represents the patterns of cooperation between countries, with color indicating different categories, circle size reflecting the number of national publications, and line thickness indicating the intensity of cooperation. It is worth noting that the United States has the most frequent cooperation with other countries, while European countries, China and Brazil have also demonstrated strong cooperation with other countries. The United States not only leads in the number of publications, with 1,003 papers, but also in the average number of citations per paper, averaging 37.7. This surpasses Brazil’s average of 23.0 and is indicative of a stronger research impact compared to China, which has an average of 46.6 citations per paper. This difference may be due to the emphasis on multi-agency collaborations, such as OPERA projects, and the higher proportion of randomized controlled trial (32% compared to 18% in China) that are more likely to be cited (4). China’s rapid growth, averaging 12 percent per year since 2015, can be attributed to increased investment in scientific research and enhanced collaboration on innovation, but its low average citation rate highlights the need to balance quantity with methodological rigour, for example, stricter adherence to CONSORT guidelines for clinical research.

### 3.3 Number of publications issued by institutions

**Figure 4:**
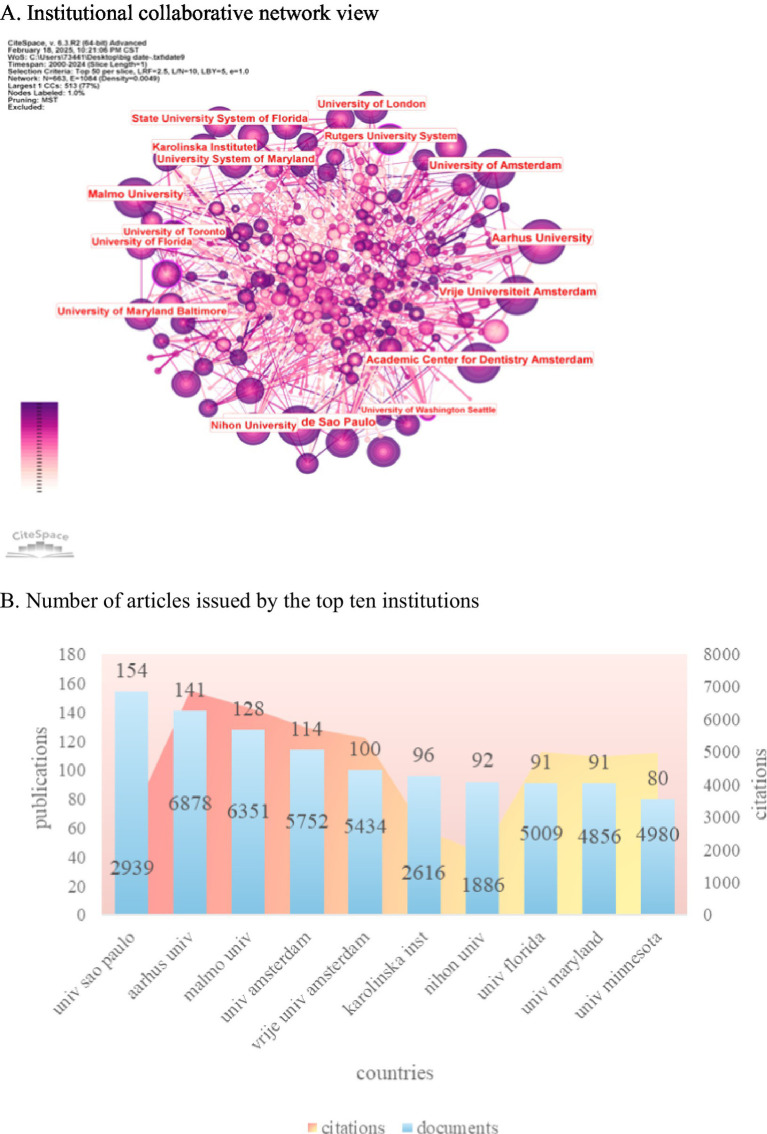
This section presents an analysis of institutional productivity and collaboration in academic publishing. **(A)** The CiteSpace network map illustrates the relationships among institutions, where node size corresponds to the number of publications, and links represent co-authorship connections. The color coding indicates time slices, with earlier publications shown in purple and more recent ones in yellow. **(B)** Additionally, a bar chart displays the top 10 most prolific institutions based on their article counts, providing a clear visual representation of their contributions to the field.

CiteSpace was utilized to examine spatial collaboration in the oral and maxillofacial region from 2000 to 2024. The analysis involved dividing the time span into individual years and identifying the top 50 institutions in each year. In Figure 4A, the size of each node corresponds to the volume of published papers, while the connections between nodes represent collaborative relationships among institutions. Figure 4B highlights the University of São Paulo in Brazil as the leading institution with 154 publications, demonstrating strong partnerships with other institutions. Following closely is Aarhus University in Denmark, which produced 141 articles. Noteworthy among the top 10 institutions are two Dutch entities, namely Amsterdam University (114 articles) and Amsterdam Free University (100 articles), as well as three American universities: Florida University (91 articles), Maryland University (91 articles), and Minnesota University (80 articles). The data reveals the prominent positions of the United States and Brazil in terms of publication output, indicating significant global influence. China excels in the number of publications by institutions and has established robust international collaborations. The upward trajectory in publication output and influence underscores the continuous growth and impact of institutions in this field. The University of São Paulo (154 articles) and Aarhus University (141 articles) ranked as the top two institutions in terms of publication output, with citation rates of 22.4 and 48.8 times per article, respectively, indicating their significant influence in the field. The high impact of Aarhus University can be attributed to its pioneering validation study of the DCTMD (110), which has been cited 650 times and established a methodological “branding effect.” The strength of the University of São Paulo is evident in that 61 percent of its publications consist of case series or cross-sectional studies. To achieve even greater impact, we recommend that future research prioritize randomized controlled trials (RCTs), actively disseminate superior resources, strengthen collaborations, and contribute to the advancement of this field.

### 3.4 Periodicals and co-cited journals

**Figure 5:**
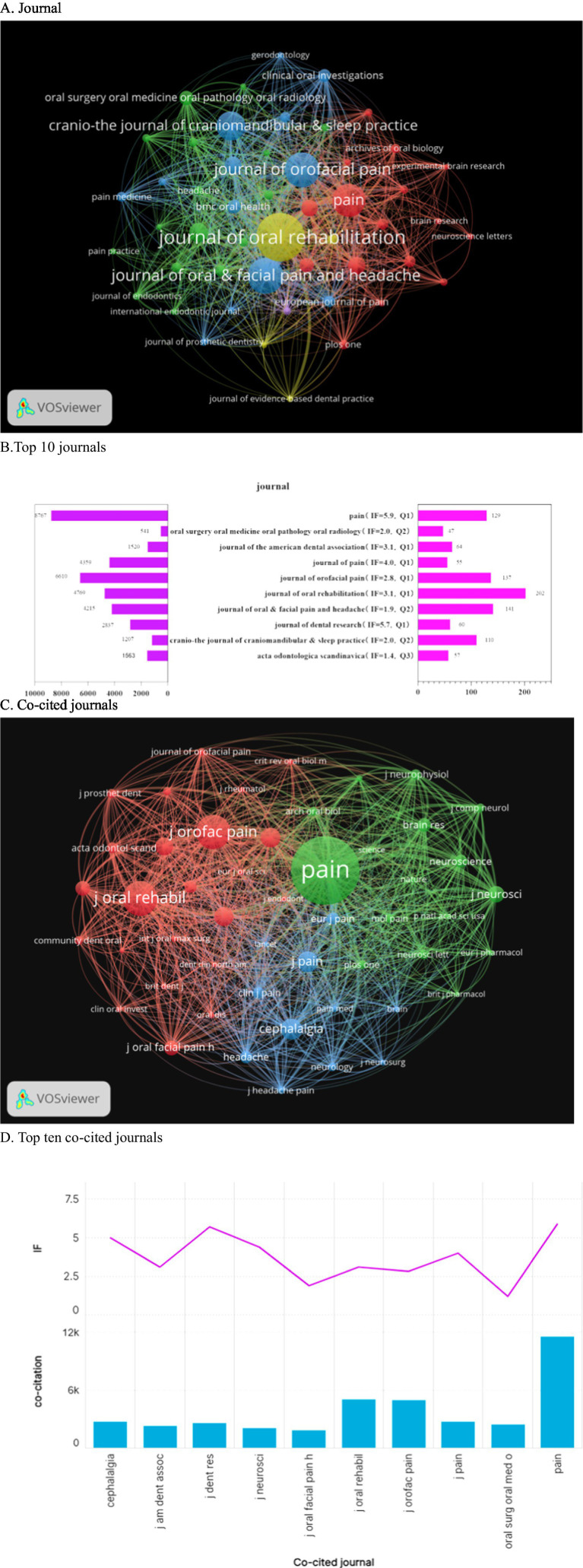
The journal landscape concerning orofacial pain is illustrated through several key aspects. **(A)** The distribution of the 627 journals that have published on orofacial pain, with a threshold of at least 15 articles, is presented. **(B)** Additionally, a ranking of the top 10 journals based on article volume is provided, along with their respective 2023 Impact Factor (IF) and total citations. **(C)** A co-citation network is depicted, showcasing three distinct clusters: (green) clinical orofacial journals, (blue) pain research journals, and (red) high-impact general science journals. **(D)** Furthermore, the top 10 co-cited journals are identified, with only “Pain” (IF = 5.9) and the “Journal of Dental Research” (IF = 5.7) surpassing an IF of 5.

A total of 627 journals have published articles on oromaxillofacial pain. When applying a threshold of 15, 50 journals meet the criteria, as depicted in Figure 5A. The Journal of Oral Rehabilitation leads in the number of published articles but ranks fourth in citations, with 4,769 citations. It exhibits the highest total connection strength, indicating extensive collaborations with other journals. Pain ranks fourth in published articles, with 129 articles, yet it garners the highest number of citations, underscoring its significant standing in the field. The top three journals by article count are the Journal of Oral Rehabilitation (*n* = 202), the Journal of Oral & Facial Pain and Headache (*n* = 141), and the Journal of Oral Pain (*n* = 137). Pain boasts the highest impact factor (IF = 5.9), followed by the Journal of Dental Research (IF = 5.7, Q1) (Figure 5B). A co-citation network graph, constructed from 50 journals with a minimum citation count of 462, reveals three distinct clusters, each denoted by a different color. The green cluster comprises prestigious journals like Science, Nature, and Pain, symbolizing the forefront and pinnacle of their respective fields. The blue cluster is centered around the Journal of Pain, while the red cluster is dedicated to studies on oral and maxillofacial pain, as illustrated in Figure 5C. Among the 12,859 journals included in the analysis, only Pain (11,529 citations) exceeded 10,000 citations. Notably, only Pain and the Journal of Dental Research boasted an impact factor exceeding 5 points, as depicted in Figure 5D. The Journal of Oral Rehabilitation (202 articles) led in quantity, but Pain (129 articles, 8,767 citations) dominated in influence (IF = 5.9), partly due to its strict peer review focusing on mechanistic depth. Co-citation analysis revealed that high-impact journals (e.g., Nature, Pain) clustered around translational research, while specialty journals (e.g., Journal of Oral & Facial Pain and Headache) focused on clinical applications. Notably, only 28% of articles in top journals reported sample size calculations, indicating a gap in methodological transparency.

### 3.5 Analysis of authors and co-cited authors

**Figure 6:**
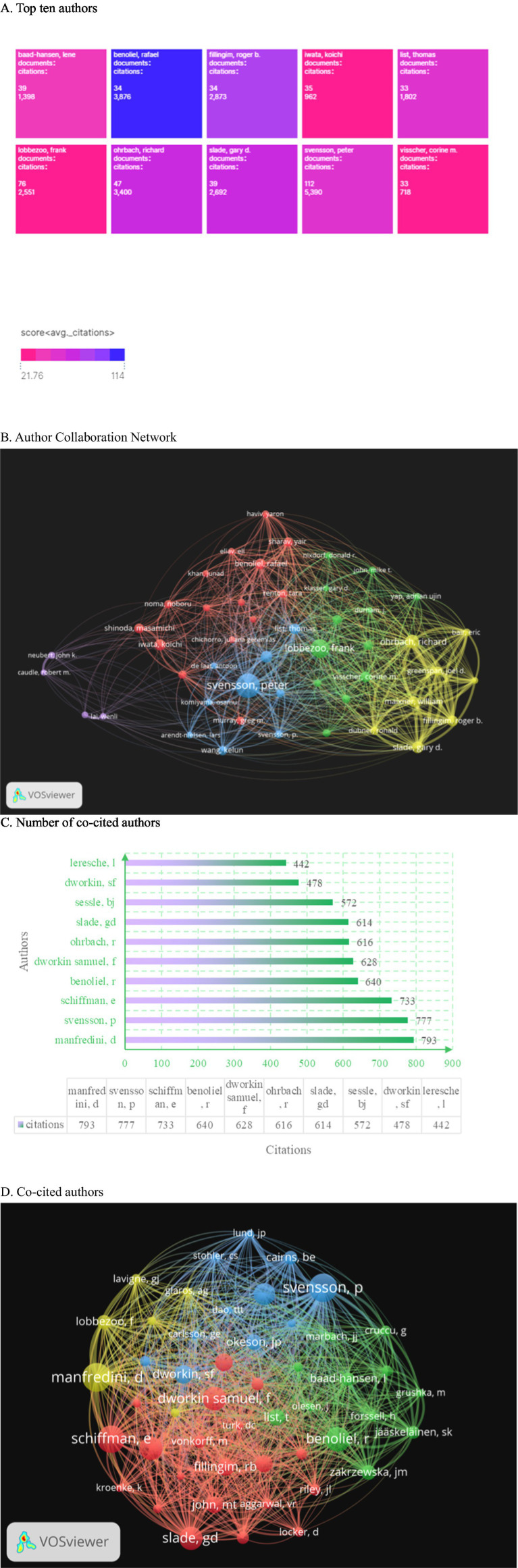
The analysis of author and co-cited author networks reveals several key insights. **(A)** The bar chart illustrates the top 10 most prolific authors in the field, highlighting their respective publication outputs. **(B)** The author collaboration map generated using VOSviewer demonstrates the strength of collaborations, with the distance between nodes representing collaboration strength and the size of each node indicating the volume of publications. **(C)** Additionally, the total citations received by the 10 most cited authors are presented, providing a quantitative measure of their influence. **(D)** Lastly, the co-cited author clusters are visually represented, with four distinct color-coded groups centered around prominent figures: Manfredini (yellow), Schiffman (red), Svensson (blue), and Benoliel (green). These visualizations collectively underscore the interconnectedness of authors and their contributions to the literature.

Among authors who published literature on oral and maxillofacial pain from 2000 to 2024, Figure 6A displays the top 10 most influential authors. Peter Svensson (*n* = 112) emerges as the most prolific author in oral and maxillofacial pain, followed by Frank Lobbezoo (*n* = 76) and Richard Ohrbach (*n* = 47). Combined with Figure 6B the author collaboration network diagram further confirms that the first two authors occupy prominent positions within the collaboration network, indicating their significant influence in this field. Figure 6C reveals that Schiffman is cited 793 times. The co-cited author network visualization diagram establishes a threshold of 164 citations per author, resulting in four clusters. The yellow cluster, with Manfredini at its core, is cited 793 times. The red cluster, centered on Schiffman, garners 733 citations. The blue cluster, led by Svensson, accumulates 777 citations. The green cluster, with Benoliel at its center, is cited 640 times as depicted in Figure 6D. Peter Svensson ranks first in both the number of published articles and citations, underscoring his significant influence in this field. The citation rates for Svensson (53.4 times per article) and Lobbezoo (55.5 times per article) were significantly higher than that of Schiffman (83.2 times per article). However, Schiffman’s elevated average is largely influenced by a single consensus document on DC/TMD published in 2014, which received 650 citations, while the remaining 46 articles averaged only 31.4 citations each. This indicates a “standout” case rather than a consistent pattern of high citations.

### 3.6 Analysis of co-cited references

**Figure 7:**
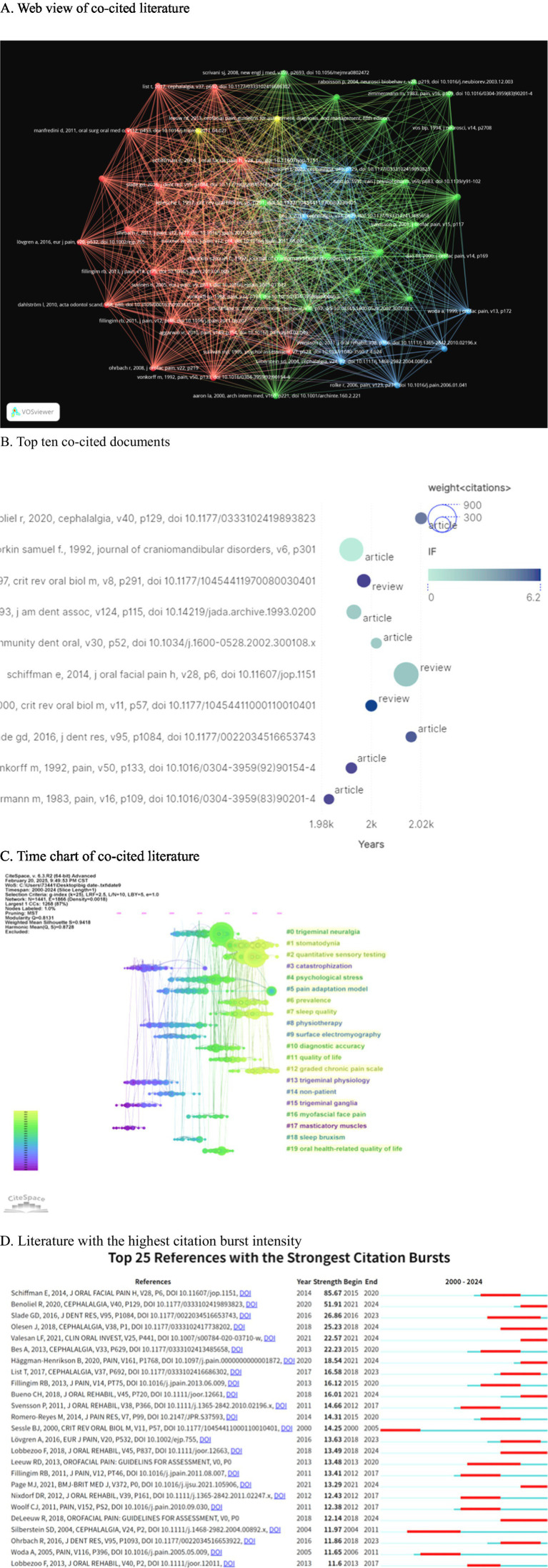
The co-citation reference analysis conducted using CiteSpace reveals several key insights into the academic landscape surrounding the topic. **(A)** The network of the 50 most co-cited references is illustrated, with node size representing citation frequency, indicating the prominence of these works in the field. **(B)** Additionally, the top 10 most cited individual papers have been identified, highlighting significant contributions to the literature. **(C)** The timeline view presents the horizontal axis as the publication year, with clusters illustrating thematic evolution from “trigeminal ganglia” (2000–2005) to “quantitative sensory testing” (2015–2024). **(D)** Furthermore, the top 25 references with the strongest citation bursts are displayed; the length of the bars indicates both the duration and intensity of these citation bursts, exemplified by Schiffman’s 2014 DC/TMD paper, which has a burst value of 85.67.

The co-cited papers visualization is created using node size and connection line strength (see Figure 7A). Figure 7B displays the top 10 most cited articles, with Schiffman et al.’s work titled “International RDC/TMD Jointly Published Diagnostic Criteria for Temporomandibular Joint Disorders (DC/TMD) in Clinical and Research Applications” having the highest number of citations, totaling 650.

Furthermore, a co-citation analysis was conducted on the timeline literature (Figure 7C). The analysis revealed that “catastrophization” (cluster 3), “trigeminal physiology” (cluster 13), “trigeminal ganglia” (cluster 15), and “predominant muscles” (cluster 17) emerged as early focal points. In the mid-term period (2005–2015), “trigeminal neuralgia” (cluster 0), “psychological stress” (cluster 4), “pain adaptation model” (cluster 5), “physiotherapy” (cluster 8), “surface electrography” (cluster 9), “non-patient” (cluster 14), “myofascial face pain” (cluster 16), “sleep bruxism” (cluster 18), and “oral health-related quality of life” (cluster 19) were identified as research hotspots. Subsequently, in the recent years (2015–2024), “stomatodynia” (cluster 1), “quantitative sensory testing” (cluster 2), “prevalence” (cluster 6), “sleep quality” (cluster 7), “quality of life” (cluster 11), and “graded chronic pain scale” (cluster 12) have emerged as popular research topics and continue to be hot spots in the realm of oral and maxillofacial pain.

In Figure 7D, we present the top 25 references with the highest citation bursts. Among these, Schiffman, E. authored “Diagnostic Criteria for Temporomandibular Disorders (DC/TMD) for Clinical and Research Applications: Recommendations of the International RDC/TMD Consortium Network*” and the Orofacial Pain Special Interest Group, with an explosion intensity of 85.67, indicating significant impact. Benoliel, R. published “Classificação Internacional de Dor Orofacial, Primeira Edição (ICOP)—versão Português Brasileiro” in 2020, with an explosion intensity of 51.91, also demonstrating substantial influence in the field. Eighty percent of the highly cited articles were published between 2010 and 2015, with nearly all of them being diagnostic or consensus documents. After 2019, there was a sharp decline in the number of highly cited articles, indicating a phenomenon referred to as “standard saturation.” Concurrently, despite a significant increase in research on IL-6, TNF-α, and other molecular studies, these topics have yet to enter the top 10 of co-citation cores. This suggests that the current research frontier is becoming disconnected from the classical knowledge base. To address the “high citation-low transformation” gap, priority should be given to research that can validate molecular mechanisms and diagnostic criteria within a closed loop.

### 3.7 Keyword and bursty keyword analysis

**Figure 8:**
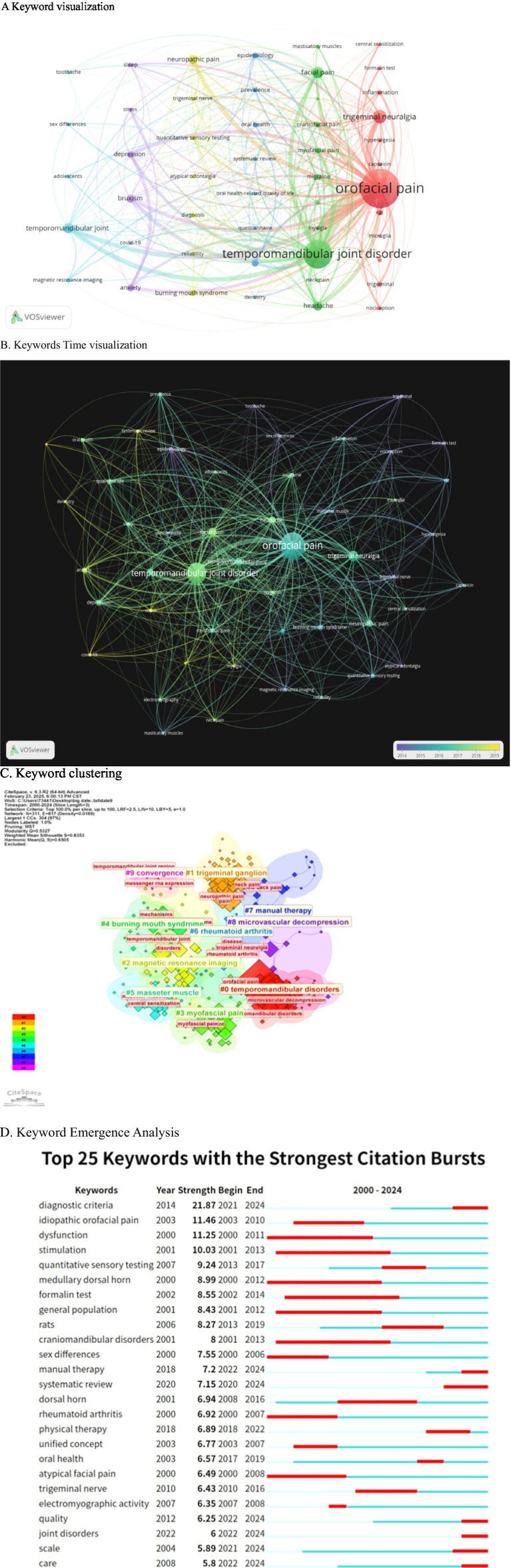
The analysis of keyword dynamics reveals significant trends in the research landscape. **(A)** The VOSviewer density map illustrates 49 high-frequency keywords, with a threshold of 24 occurrences, where warmer colors indicate a higher co-occurrence density. **(B)** The overlay visualization highlights that yellow keywords, representing the most recent research (2022–2024), include terms such as “COVID-19” and “sleep quality.” **(C)** The keyword time-line clusters depict a transition from earlier mechanistic terms, such as “dorsal horn” and “formalin test,” to contemporary clinical-translational terms, including “diagnostic criteria” and “manual therapy.” **(D)** Additionally, the top 20 keywords exhibiting the strongest emergence bursts indicate that the longest current burst is associated with “systematic review.”

Keyword frequency and link strength were assessed using VOS software. When the minimum keyword occurrence was set at 24, a total of 5,537 keywords were identified. Subsequently, with a threshold of 24, 49 keywords were obtained. The most prevalent keyword was “orofacial pain” (1,336 occurrences), followed by “temporomandibular joint disorder” (822), “trigeminal neuralgia” (237), “facial pain” (175), and “temporomandibular joint” (147) (Figure 8A). In Figure 8B yellow highlights current research hotspots including “stress,” “systematic review,” “dentistry,” “depression,” “COVID-19,” and “oral health-related quality of life.” “orofacial pain” emerged as a consistent hot topic throughout the entire period and exhibited the highest total link strength among all keywords.

This knowledge map comprises 817 keywords from oral and maxillofacial literature collected from the Web of Science Database between 2000 and 2024, analyzed using CITESPACE software (Figure 8C). The color clusters represent core research directions: Cluster #0 focuses on the diagnosis, pain mechanisms, and surgical intervention of temporomandibular disorders (TMD); Cluster #1 investigates the role of the trigeminal ganglion in oral and maxillofacial pain and associated symptoms; Cluster #2 discusses the application of magnetic resonance imaging in diagnosing temporomandibular joint diseases; Cluster #3 explores the mechanisms underlying masseter myofascial pain and central sensitization; Cluster #4 emphasizes the etiology and mechanisms of burning mouth syndrome; Cluster #5 examines myofascial pain resulting from masseter dysfunction; Cluster #6 addresses the involvement of the temporomandibular joint in rheumatoid arthritis; Cluster #7 looks at the application of manipulation in related diseases; and Cluster #9 investigates the molecular mechanisms of pain signaling in oral and maxillofacial contexts. By concentrating on oral and maxillofacial pain and integrating anatomy, imaging, molecular biology, and clinical disciplines, this study presents a closed loop of “Diagnosis-Mechanism-Intervention,’’ which supports the identification of research hotspots, the exploration of knowledge gaps, and the optimization of diagnosis and treatment strategies.

In Figure 8D, the following terms are examined during the early phase: “dysfunction,” “stimulation,” “idiopathic oral pain,” “meditative dorsal horn,” “formalin test,” “general population,” “cranidibular disorders,” “sex differences,” “rheumatoid arthritis,” and “atypical facial pain.” In the intermediate phase, the analysis includes “quantitative sensory testing,” “rats,” “dorsal horn,” “pathological therapy,” “unified concept,” “oral health,” “trigeminal nerve,” and “electromyographic activity,” as well as subsequent topics such as “diagnostic criteria,” “manual therapy,” “systematic review,” “quality,” “joint disorders,” “scale,” and “care.”

**Figure 9:**
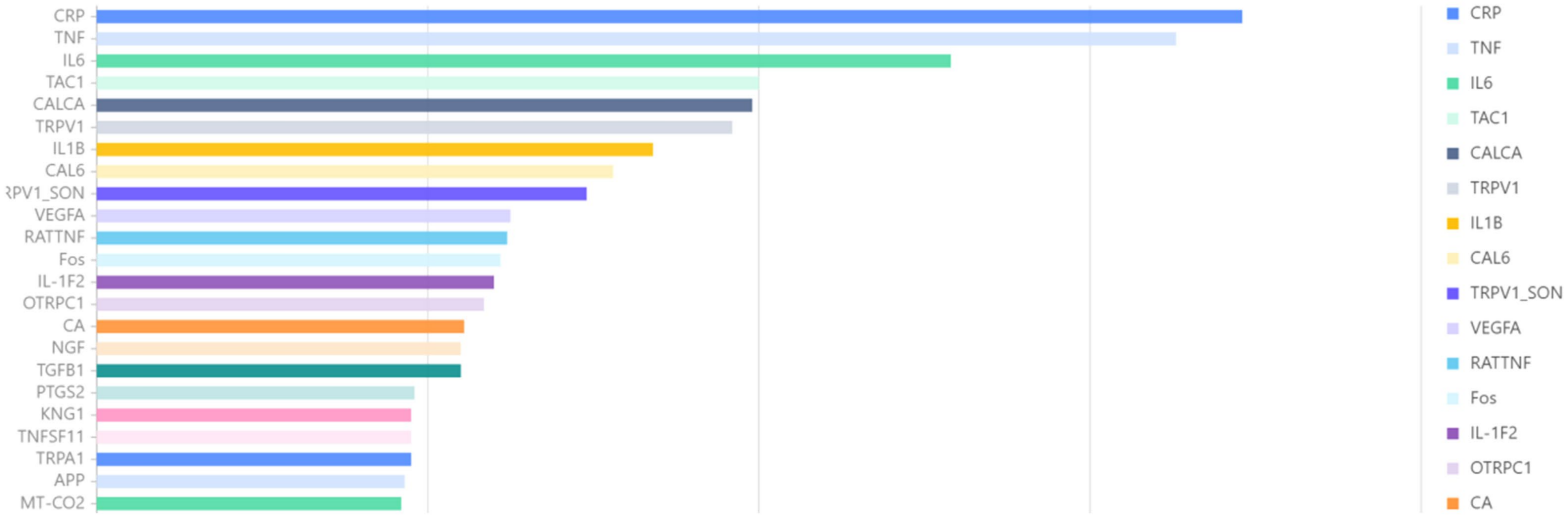
Association gene analysis. This study examines gene-level trends associated with orofacial pain, utilizing a heat map generated through BioBERT extraction to illustrate the 30 most frequently mentioned genes from 2000 to 2024. The color intensity of the heat map corresponds to the proportional annual frequency of these genes. Notably, CRP, TNF, and IL-6 consistently dominate the data across all years, with IL-6 exhibiting a significant surge post-2015, indicating its potential as a translational target for research and therapeutic interventions.

While the bibliometric corpus captures clinical and behavioral trends, it does not reveal the molecular substrates that drive these trends. Therefore, we have incorporated a targeted layer of gene entity extraction. In the genetic analysis presented in Figure 9, a total of 24,600 articles published between 2000 and 2024 were identified by searching for keywords related to orofacial pain, facial pain, maxillofacial pain, craniofacial pain, toothache, and jaw pain. Gene entities were extracted and statistically analyzed from the abstracts of these articles using the BioBERT biomedical language model. Among the analyzed genes, CRP was the most frequently mentioned in the literature, appearing in 346 articles, followed by TNF (326 articles) and IL6 (258 articles). This quantification helps to elucidate which inflammatory or neuromodulatory pathways (IL-6, TNF, CRP) are concurrently elevated alongside keyword bursts such as “central sensitization” or “systematic review.” Furthermore, it is beneficial to provide a translation bridge if a gene is cited more rapidly than its clinical descriptors, indicating readiness to transition applications from the laboratory to the clinic. This approach also facilitates researchers in cross-validating connections between gene trajectories and the citation surges of DC/TMD diagnostic criteria to examine whether mechanisms and pathological progression are synchronized. This two-scale design preserves the integrity of the macro-level narrative while providing funders and steering groups with a data-driven snapshot of molecular momentum.

## 4 Discussion

### 4.1 Geographical patterns

We systematically analyzed 3,372 papers on oral and maxillofacial pain published between 2000 and 2024 using CiteSpace and VOSviewer software with data sourced from the Web of Science database. In Figure 3A, the distribution of national publications is depicted, with the United States (1003), China (302), and Brazil (424) emerging as the top three contributors. These countries exhibit close collaboration with numerous other nations. Particularly, the United States engages extensively in academic exchanges and demonstrates significant global influence, This reflects the strength of its scientific research. China, characterized by a burgeoning annual publication output and collaborative partnerships with various countries, is experiencing a notable increase in influence over time.

Among the top 10 institutions worldwide in terms of the number of published papers (Figure 4), 90% are from developed countries. Notably, the United States contributes 30% of these publications, underscoring its significant role in advancing academic research in this field. The University of São Paulo in Brazil leads with 154 publications, yet its citation rate is relatively low, indicating a need to enhance the quality of its scientific output. Aarhus University in Denmark closely follows with 141 articles, ranking second in publication volume but first in citations, amassing a total of 6,878 citations at an average of 48.78 citations per document. This highlights the institution’s exceptional publication quality and its pivotal position in the field. While China ranks in the global top 10 for the number of publications, its institutions do not feature among the top 10 globally. This suggests a dispersed presence of Chinese institutions with limited inter-institutional collaboration. Moving forward, there is a pressing need to bolster collaboration efforts and prioritize the enhancement of scientific research quality in China.

The journal of Oral Rehabilitation has the highest number of articles published, with 202 citations and 8,767 total citations for pain, underscoring its significant standing in oral and maxillofacial pain research. Figure 6 reveals Svensson, Peter as the leading author in oral and maxillofac.

### 4.2 Keyword trends

Analysis of early-stage terms reveals fundamental theories, classical studies, and key trends in the field of oral and maxillofacial pain. Examining mid-term emerging terms uncovers current focal points and leading-edge developments, guiding scholars in aligning their research with prevailing trends and fostering a sharp research acumen. Late-stage emergent terms mirror the progression of trending topics, enabling timely adjustments to research inquiries and orientations to advance relevant technological developments. Evaluating post-emergence research trends offers insights into the research significance and potential of hot topics in oral and maxillofacial pain research.

Previous studies have linked temporomandibular dysfunction (42), sleep disorder (43), social disorder (44), myofascial disorder (45), and styloid process elongation to the onset or exacerbation of oral and maxillofacial pain (46), which can be influenced by psychological, anatomical, and environmental factors. Variations in oral and maxillofacial pain thresholds and tolerances have been observed among different populations and in response to mechanical, temperature, and electrical stimuli (47). Stimulation techniques can be used to assess oral and maxillofacial sensitivity and are integral to treatments like transcranial stimulation therapy and electrical stimulation-induced muscle regulation (48–50). Idiopathic facial pain, characterized by facial pain without a discernible cause (51, 52) such as trauma, infection, or tumor, represents a pain syndrome of unknown etiology, potentially linked to neurological disorders (53), vascular compression, genetic factors (54), and immune responses (55). Atypical facial pain is a type of facial pain with an unknown etiology that does not fit into any recognized facial pain syndrome category (56, 57). It typically presents as vague, challenging to articulate pain in the facial, cranial, or cervical regions, resembling idiopathic pain. Studies have shown a prevalence of pain attacks in the general population, with notable gender variations (58–60). Research indicates a connection between the medullary dorsal horn and orofacial pain (61, 62). The medullary dorsal horn serves as a crucial processing center for sensory input from the trigeminal nerve, where pain signals from the oral and maxillofacial areas are initially received and interpreted. Various factors can contribute to orofacial pain, including oral pathologies (e.g., pulpitis, periapical periodontitis, pericoronitis of wisdom teeth), temporomandibular joint disorders, and neuropathic conditions (e.g., trigeminal neuralgia). Early-stage rheumatoid arthritis has been associated with pain interactions (63, 64), potentially affecting the oral and maxillofacial regions. Rheumatoid arthritis, an autoimmune disorder, not only impacts limb joints but can also involve the temporomandibular joint, leading to symptoms like pain, swelling, and restricted movement. The condition involves the immune system attacking synovial tissue, cartilage, and other orofacial structures, contributing to the experience of pain.

Research on children’s oral health has gained significant attention in the medium term, emphasizing its importance (65). Studies on the temporomandibular joint and oral health-related indices, such as the quantitative sensory test (QST), have emerged as crucial methods for evaluating sensory function in diagnosing oral and maxillofacial pain (66, 67). QST enables the measurement of various sensory thresholds, including temperature perception and pain, providing valuable insights into the sensory status of the oral and maxillofacial region (68). Oral and maxillofacial pain is intricately linked to electromyography (EMG) activity. Normal conditions exhibit specific EMG activity patterns in the muscles of the oral and maxillofacial region. However, the onset of oral and maxillofacial pain often leads to alterations in EMG activity in the affected muscles. For instance, in cases of temporomandibular joint disorder causing oral and maxillofacial pain, the electromyography activity of masticatory muscles (such as the masseter muscle and temporal muscle) may display heightened potential and irregular muscle activity. Analyzing EMG activity proves beneficial in diagnosing muscle-related origins of oral and maxillofacial pain and evaluating muscle functional status (69, 70). Various physical therapies are employed for oral and maxillofacial pain management. Warm compresses improve local blood flow, alleviate muscle spasms, and mitigate pain. Conversely, cold compress application during the onset of pain can diminish swelling and decrease nerve ending sensitivity. Massage techniques are employed by professionals to alleviate tension in oral and maxillofacial muscles, such as masticatory and temporal muscles, thereby improving muscle flexibility (71, 72). Ultrasound therapy is utilized to penetrate deep tissues, leveraging its warmth and mechanical effects to alleviate pain. Additionally, transcutaneous electrical nerve stimulation is employed to activate nerve fibers and induce *in vivo* caffeine release for pain reduction (73, 74). Oral and maxillofacial pain nociceptor activation can stem from various factors, triggering pain signal transmission and central sensitization processes. This phenomenon is associated with a wide array of causes, including oral conditions like caries, pulpitis, apicitis, and pericoronitis of wisdom teeth. Primary nerve diseases, such as trigeminal and glossopharyngeal neuralgia, along with musculoskeletal disorders like muscle dysfunction, temporomandibular dysfunction, and bruxism (75), share a common pathogenesis. This includes the activation of nociceptor nerve endings, the transmission of pain signals along nerve fibers (76) to trigeminal subnucleus caudalis, and subsequently to the cerebral cortex via the spinothalamic tract. Prolonged exposure to pain can induce plastic changes in the central nervous system, resulting in heightened sensitivity of trigeminal nerve to pain signals. This heightened sensitivity can lead to a robust pain response even with minor stimulation, thereby playing a crucial role in chronic oral and maxillofacial pain (77). The trigeminal nerve, the fifth pair of cranial nerves, is a mixed nerve comprising the ophthalmic, maxillary, and mandibular branches. It governs the tactile, pain, and temperature sensations of the oral and maxillofacial regions. Its association with oral and maxillofacial pain can manifest as secondary primary trigeminal neuralgia and can transmit pain from other oral and maxillofacial conditions such as pulpitis, periapical periodontitis, periodontitis, and temporomandibular joint disorders. The trigeminal nerve is intricately involved in pain modulation and harbors various neurotransmitters and modulators like substance P and calcitonin gene-related peptide.

Currently, research primarily focuses on the molecular mechanisms (78), neuroplasticity (79), and neuroimmune interactions (80) underlying oral and maxillofacial pain. The diagnostic criteria for such pain are multifaceted and vary depending on the underlying cause. Typically, a comprehensive approach is adopted, involving a thorough collection of medical history, clinical examinations encompassing oral, maxillofacial, and neurological assessments, imaging techniques like X-ray, CT, and MRI, as well as additional tests such as pulp vitality and electromyography examinations (23, 81). Treatment of oral and maxillofacial pain often involves a combination of therapies, including physical interventions (82, 83), manual techniques like massage (84), exercise regimens including targeted muscle-stretching and controlled chewing exercises, occlusal treatments like splints and orthodontic interventions, injection therapies like joint cavity injections (85) and nerve blocks, and psychotherapeutic approaches such as cognitive-behavioral therapy (86). In recent years, there has been a growing focus on the systematic analysis of oral and maxillofacial pain diseases, aiming to investigate various aspects such as incidence, etiology, treatment, mechanisms, and impacts (65, 87, 88). Oral and maxillofacial pain can be attributed to joint disorders, including conditions like masticatory muscle disorders caused by prolonged unilateral chewing and increased mental stress, leading to excessive tension in the masticatory muscles, resulting in pain. Temporomandibular joint disorders manifest with symptoms such as joint snapping, pain, and restricted mouth opening. Additionally, oral diseases like periodontitis and pulpitis can also induce pain when they affect the surrounding tissues. Current research by scholars is centered on the environmental factors, treatment modalities, and pathogenesis of these diseases (89–91). Various pain assessment scales, validated tools such as the 0–10 visual analogue scale (VAS) and the 11-point numerical rating scale (NRS) are routinely used to quantify pain intensity and distinguish between different disease conditions (92, 93). The presence of oral and maxillofacial pain significantly impacts the quality of life of patients (94). Simultaneously, the quality of life plays a crucial role in the progression of oral and maxillofacial pain. Monitoring changes in patients’ quality of life is closely associated with the onset, advancement, and management of these diseases (95, 96). Effective nursing care is vital for daily pain management, encompassing practices such as ensuring adequate rest, consuming softer foods to prevent exacerbation of pain, emotional self-regulation for psychological well-being, and localized application of heat compresses to enhance blood circulation (97–99).

Building on existing bibliometric foundations in dentistry and pain research, our analysis addresses a critical blind spot: prior studies have treated orofacial pain either as a regional subset of chronic pain or as an ancillary outcome of dental disease, thereby overlooking its unique position at the intersection of dentistry, pain science, and psychology. We demonstrate, for the first time, that orofacial pain constitutes an independent, transdisciplinary field. First, by foregrounding oral-specific mechanisms—such as the trigeminal ganglion, the temporomandibular joint, and their bidirectional crosstalk with the central nervous system (CNS)—we extend pain bibliometrics beyond spinal pathways and single-discipline silos. Second, we recenter pain as the primary research object: diagnostic criteria based on the International Classification of Orofacial Pain (ICOP) and botulinum toxin paradigms challenge the disease-centric narrative prevalent in dental literature, while molecular pathways (e.g., TNF, IL-6) and psychosocial factors emerge as core drivers of pain chronification. Finally, integrating BioBERT gene-entity extraction dynamically couples macro-level citation trends with micro-level molecular targets (e.g., IL-6 in temporomandibular joint pain), offering a new methodological toolkit for identifying translational opportunities. Collectively, these advances push general pain and dental bibliometrics toward a domain-specific, biopsychosocial framework.

This study presents the inaugural bibliometric analysis of oral and maxillofacial pain, offering a comprehensive overview of its development and research landscape. The findings aim to aid scientific research decision-making processes and serve as a valuable reference for various aspects within the field, including collaboration choices, journal selection, and manuscript preparation. By identifying emerging research trends, highlighting key areas of interest, and outlining future directions of study in oral and maxillofacial pain, this analysis is instrumental in guiding discipline planning and enhancing scholars’ comprehension of the academic research standards and advancements in this domain.

However, there are notable deficiencies in the current research. The database utilized is limited in scope, warranting a comprehensive search across multiple databases. The large volume of retrieved records raises the risk of inadvertent omissions, underscoring the importance of standardizing retrieval formulas and procedures. Disparities in research output across different regions highlight the need for enhanced inter-regional collaboration to facilitate knowledge exchange and collaborative advancement. Furthermore, the exploration of specific research avenues, such as delving into the intricate mechanisms underpinning the relationship between distinct etiologies and pain, remains inadequate, signaling a necessity to augment investments in these promising domains moving forward.

### 4.3 Future recommendations

This bibliometric map not only charts the research landscape but also translates data into actionable insights. By highlighting “diagnostic criteria” and “manual therapy” as dominant yet evolving hotspots, we identify priority areas for funding: longitudinal validation of ICOP-2020 subtypes and mechanism-driven trials comparing manual therapy to botulinum toxin. The persistent citation burst of Schiffman’s DC/TMD criteria (burst = 85.67) underscores the necessity for training curricula that incorporate these standardized tools into dental and neurology residency programs. Furthermore, the underrepresentation of quality-of-life endpoints in high-impact journals—where only 28% report sample-size calculations—indicates that future clinical guidelines should mandate the inclusion of patient-reported outcome measures alongside traditional pain scores, ensuring that resource allocation aligns with patient-centered care rather than solely with publication metrics.

Currently, there are several promising biomarkers, including salivary cortisol in sialomics, a multiplex panel encompassing DHEA, neuropeptide Y, IL-6, TNF-α, and the miR-146 and let-7 families, as well as a panel targeting the salivary gland for real-time monitoring of orofacial pain (100). In circulating microRNAs, leader miR-34a-5p and miR-331-3p serve as early, non-invasive indicators of TMJ-related chronic pain, following the precedent set by depression panels, thereby facilitating early diagnosis and treatment of orofacial pain (101). In immunometabolic heterozygotes, the combination of high-sensitivity CRP with plasma diacetamine (a doxorubicin-reactive metabolite) predicts treatment refractoriness in the trigeminal nerve (102). With the rise of AI, its application in this field has become increasingly widespread, including pain type classifiers, training gradient boosting models on longitudinal saliva miRNA and QST data, and enhancing the accuracy of classification results to predict the transition from acute to chronic orofacial pain (103). The visualization of Shap values in interpretable AI dashboards is integrated into electronic health record plug-ins, allowing clinicians to identify which biomarkers or psychological covariates influence each risk score (104). In joint learning, privacy-preserving machine learning is conducted between multicenter TMJ registries (USA Opera, Denmark TMD case) without sharing raw patient data (105). It is essential to utilize richer databases and bibliometric research methods, such as enhanced data sources, the use of PubMed’s open-access subset, the European PMC preprint, and clinicaltrials.gov to supplement Web of Science, in order to capture grey literature and negative results (106). Linking genes, devices, and psychological scales through BioBERT and UMLS in entity extraction promotes keyword counting for automated concept recognition (107). At the temporal granularity level, the R package bibliometrics was employed to transition from annual slices to quarterly snapshots, capturing rapidly evolving research or policy shifts related to orofacial pain (108). Certainly, at the validation level, we should cross-map emerging terms with existing systematic review platforms such as Cochrane Pain and Prospero to flag underrepresented studies and avoid duplication (109).

In the future, more in-depth studies are needed to explore the interactions within neuro-immune-endocrine networks, the application of biomarkers for personalized diagnosis and treatment plans, and the use of organoid models or artificial intelligence to predict pain progression. These advancements offer new insights into the mechanisms and management of oral and maxillofacial pain; however, significant challenges remain for interdisciplinary collaboration and clinical application. In the future, there is a need to delve deeper into the interplay of neuro-immune-endocrine networks, create personalized diagnostic and treatment plans utilizing biomarkers, and forecast the progression of pain using organoid models or artificial intelligence. These advancements offer fresh perspectives on the mechanisms and management of oral and maxillofacial pain; however, interdisciplinary cooperation and clinical application pose significant challenges.

## 5 Conclusion

After a quarter-century of accumulating global scholarly research, this bibliometric analysis positions orofacial pain as an emerging interdisciplinary frontier rather than merely a subdiscipline of dental or general pain science. By integrating 3,372 literature records into a dynamic evolution of knowledge map, it reveals several key transitions for researchers. Among these, the three most important aspects are: (1) a shift from symptom-anchored investigations to mechanism-centered investigations, indicated by the predominance of trigeminal neuron-specific pathways and IL-6/TNF-α signaling; (2) the academic influence of researchers and institutions has transitioned from a “North-led” pattern to form multi-center, albeit uneven, collaboration networks that focus more on the mechanistic depth of research rather than the volume of research. It is essential to strengthen academic exchanges and cooperation, grasp the research frontier hotspots, and facilitate the transition from clinical research to clinical application; (3) the paradigmatic expansion of therapeutic discourse from procedural intervention to an integrated bio-psycho-social model is reflected in the continued surge in citations related to the DC/TMD criteria and manual therapeutic evidence. These macro trends collectively highlight a core need: future research progress will no longer depend on the simple accumulation of case series. Instead, we must build coordinated, federated data ecosystems that integrate technologies such as saliva multiomics and interpretable artificial intelligence, while leveraging existing systematic review infrastructures to avoid cognitive redundancy, identify the best practices, and discard the ineffective ones. In summary, this study constructs a new framework for a “Precision Medicine Methodology Proving Ground” for oral and facial pain research. The implementation of this framework necessitates the establishment of an interoperability registry system, the adoption of privacy-protective data analysis methods, and the development of training systems for clinical scientists to dismantle the barriers posed by traditional departmental structures.

## Data Availability

The original contributions presented in the study are included in the article/supplementary material, further inquiries can be directed to the corresponding author.

## References

[1] Benoliel R Svensson P Evers S Wang SJ Barke A Korwisi B . The IASP classification of chronic pain for ICD-11: chronic secondary headache or orofacial pain. Pain. (2019) 160:60–8. doi: 10.1097/j.pain.0000000000001435, PMID: 30586072

[2] International Headache Society. International Classification of Orofacial Pain, 1st edition (ICOP). Cephalalgia. (2020) 40:129–221. doi: 10.1177/0333102419893823 32103673

[3] Alshammari SS Amin S Siddiqui AA Malik YR Alshammari AF Amin J. An evidence-based treatment of myofascial pain and myofascial trigger points in the maxillofacial area: a narrative review. Cureus. (2023) 15:e49987. doi: 10.7759/cureus.49987, PMID: 38179392 PMC10766389

[4] Slade GD Ohrbach R Greenspan JD Fillingim RB Bair E Sanders AE . Painful temporomandibular disorder: decade of discovery from OPPERA studies. J Dent Res. (2016) 95:1084–92. doi: 10.1177/0022034516653743, PMID: 27339423 PMC5004239

[5] Kaur S Hickman TM Lopez-Ramirez A McDonald H Lockhart LM Darwish O . Estrogen modulation of the pronociceptive effects of serotonin on female rat trigeminal sensory neurons is timing dependent and dosage dependent and requires estrogen receptor alpha. Pain. (2022) 163:e899–916. doi: 10.1097/j.pain.0000000000002604, PMID: 35121697 PMC9288423

[6] Seol SH Chung G. Estrogen-dependent regulation of transient receptor potential vanilloid 1 (TRPV1) and P2X purinoceptor 3 (P2X3): implication in burning mouth syndrome. J Dent Sci. (2022) 17:8–13. doi: 10.1016/j.jds.2021.06.007, PMID: 35028015 PMC8739235

[7] Cruz D Monteiro F Paço M Vaz-Silva M Lemos C Alves-Ferreira M . Genetic overlap between temporomandibular disorders and primary headaches: a systematic review. Jpn Dent Sci Rev. (2022) 58:69–88. doi: 10.1016/j.jdsr.2022.02.002, PMID: 35242249 PMC8881721

[8] Kobayashi S Osaki H Kato S Kobayashi K Kobayashi M. Regulation of nociception by long-term potentiation of inhibitory postsynaptic currents from insular cortical parvalbumin-immunopositive neurons to pyramidal neurons. Pain. (2025) 166:1823–35. doi: 10.1097/j.pain.0000000000003518, PMID: 39841043

[9] Tang C Gomez K Chen Y Allen HN Hestehave S Rodríguez-Palma EJ . C2230, a preferential use- and state-dependent CaV2.2 channel blocker, mitigates pain behaviors across multiple pain models. J Clin Invest. (2024) 135:e177429. doi: 10.1172/JCI177429, PMID: 39656529 PMC11827853

[10] Machado TMMM Aquino IG Franchin M Zarraga MO Bustos D Spada FP . Novel apocynin regulates TRPV1 activity in the trigeminal system and controls pain in a temporomandibular joint neurogenic model. Eur J Pharmacol. (2024) 985:177093. doi: 10.1016/j.ejphar.2024.177093, PMID: 39489280

[11] Steel SJ Robertson CE. First bite syndrome: what neurologists need to know. Curr Pain Headache Rep. (2021) 25:31. doi: 10.1007/s11916-021-00950-7, PMID: 33761012

[12] Tian L Li XH Zhao YL Yi HY Liu XR Yao R . DNMT3a downregulation triggered upregulation of GABA receptor in the mPFC promotes paclitaxel-induced pain and anxiety in male mice. Adv Sci. (2025) 12:e2407387. doi: 10.1002/advs.202407387, PMID: 39679872 PMC11791956

[13] Islam J Rahman MT Ali M Kim HK Kc E Park YS. Optogenetic inhibition of ventrolateral orbitofrontal cortex astrocytes facilitates ventrolateral periaqueductal gray glutamatergic activity to reduce hypersensitivity in infraorbital nerve injury rat model. J Headache Pain. (2025) 26:41. doi: 10.1186/s10194-025-01977-6, PMID: 39994518 PMC11854010

[14] Qin W Zhang Z Yan J Han X Niu LN Jiao K. Interaction of neurovascular signals in the degraded condylar cartilage. Front Bioeng Biotechnol. (2022) 10:901749. doi: 10.3389/fbioe.2022.901749, PMID: 35573252 PMC9099211

[15] Gomez K Duran P Tonello R Allen HN Boinon L Calderon-Rivera A . Neuropilin-1 is essential for vascular endothelial growth factor A-mediated increase of sensory neuron activity and development of pain-like behaviors. Pain. (2023) 164:2696–710. doi: 10.1097/j.pain.0000000000002970, PMID: 37366599 PMC10751385

[16] Yue WWS Yuan L Braz JM Basbaum AI Julius D. TRPV1 drugs alter core body temperature via central projections of primary afferent sensory neurons. eLife. (2022) 11:e80139. doi: 10.7554/eLife.80139, PMID: 35968676 PMC9377796

[17] Baggio DF Gambeta E Souza IA Huang S Zamponi GW Chichorro JG. Ca3.2 T-type calcium channels contribute to CGRP- induced allodynia in a rodent model of experimental migraine. J Headache Pain. (2024) 25:219. doi: 10.1186/s10194-024-01921-0, PMID: 39695919 PMC11656763

[18] Seweryn P Waliszewska-Prosol M Straburzynski M Smardz J Orzeszek S Bombala W . Prevalence of central sensitization and somatization in adults with temporomandibular disorders-a prospective observational study. J Oral Facial Pain Headache. (2024) 38:33–44. doi: 10.22514/jofph.2024.037 39800954 PMC11810652

[19] Seweryn P Waliszewska-Prosol M Petrasova A Bort M Seweryn M Straburzynski M . Central sensitisation, anxiety and depressive symptoms in patients with chronic masticatory muscle pain. J Oral Rehabil. (2025). doi: 10.1111/joor.70007, PMID: 40574454

[20] Song QX Zhang YY Li YL Liu F Liu YJ Li YK . The crucial role of NR2A mediating the activation of satellite glial cells in the trigeminal ganglion contributes to orofacial inflammatory pain during TMJ inflammation. Neuropharmacology. (2024) 261:110173. doi: 10.1016/j.neuropharm.2024.110173, PMID: 39357737

[21] Li YL Zhang YY Song QX Liu F Liu YJ Li YK . N-methyl-D-aspartate receptor subunits 2A and 2B mediate connexins and pannexins in the trigeminal ganglion involved in orofacial inflammatory allodynia during temporomandibular joint inflammation. Mol Neurobiol. (2025) 62:1247–65. doi: 10.1007/s12035-024-04291-5, PMID: 38976127

[22] Darnall BD Abshire L Courtney RE Davin S. Upskilling pain relief after surgery: a scoping review of perioperative behavioral intervention efficacy and practical considerations for implementation. Reg Anesth Pain Med. (2025) 50:93–101. doi: 10.1136/rapm-2024-105601, PMID: 39909552 PMC11877026

[23] Musella G Canfora F Caponio VCA Vardas E Kouri M Nikitakis N . Oral dysaesthetic and perceptual disorder, a distinct subset of chronic orofacial pain without burning symptoms: a case-control study. J Oral Rehabil. (2025) 52:651–66. doi: 10.1111/joor.13945, PMID: 39871665 PMC12037929

[24] Piriyaprasath K Hasegawa M Iwamoto Y Kamimura R Yusuf ASH Fujii N . Effects of treadmill running on anxiety- and craniofacial pain-like behaviors with histone H3 acetylation in the brain of mice subjected to social defeat stress. PLoS One. (2025) 20:e0318292. doi: 10.1371/journal.pone.0318292, PMID: 39869606 PMC11771924

[25] Nascimento GC De Paula BB Gerlach RF Leite-Panissi CRA. Temporomandibular inflammation regulates the matrix metalloproteinases MMP-2 and MMP-9 in limbic structures. J Cell Physiol. (2021) 236:6571–80. doi: 10.1002/jcp.30341, PMID: 33611790

[26] Zhao R Ye Z Lv X Li Z Xiong X. Imaging brain networks: insights into mechanisms of temporomandibular disorders. J Dent Res. (2025) 104:380–8. doi: 10.1177/00220345241302046, PMID: 39876597

[27] Mazzitelli M Ponomareva O Presto P John J Neugebauer V. Impaired amygdala astrocytic signaling worsens neuropathic pain-associated neuronal functions and behaviors. Front Pharmacol. (2024) 15:1368634. doi: 10.3389/fphar.2024.1368634, PMID: 38576475 PMC10991799

[28] Cheema S Lagrata S Rantell KR Ahmed M Kamourieh S Matharu MS. OnabotulinumtoxinA for primary new daily persistent headache and comparison to chronic migraine. Cephalalgia. (2025) 45:3331024251317448. doi: 10.1177/03331024251317448, PMID: 39924901

[29] Chisini LA Pires ALC Poletto-Neto V Damian MF Luz MS Loomans B . Occlusal splint or botulinum toxin-a for jaw muscle pain treatment in probable sleep bruxism: a randomized controlled trial. J Dent. (2024) 151:105439. doi: 10.1016/j.jdent.2024.105439, PMID: 39510242

[30] Thepsoparn M Anukoolwittaya P Toeypromthong P Thanaboriboon C. Efficacy and safety profile of onabotulinum toxin-A injection at sphenopalatine ganglion in trigeminal neuralgia: a prospective observational study. J Headache Pain. (2024) 25:210. doi: 10.1186/s10194-024-01926-9, PMID: 39623300 PMC11613643

[31] Ryu S Zhang J Simoes R Liu X Guo Z Feng L . Regulatory T cells require peripheral CCL2-CCR2 signaling to facilitate the resolution of medication overuse headache-related behavioral sensitization. J Headache Pain. (2024) 25:197. doi: 10.1186/s10194-024-01900-5, PMID: 39528947 PMC11555869

[32] Orzeszek S Martynowicz H Smardz J Wojakowska A Bombała W Mazur G . Assessment of sleep quality in patients with orofacial pain and headache complaints: a polysomnographic study. Dent Med Probl. (2024) 61:549–62. doi: 10.17219/dmp/177008, PMID: 38832763

[33] Orzeszek S Martynowicz H Smardz J Kresse-Walczak K Wojakowska A Bombała W . Assessment of the relationship between sleep bruxism, reported pain and headache, selected health factors, and general health conditions among temporomandibular disorder patients: a preliminary report. Dent Med Probl. (2025) 62:393–9. doi: 10.17219/dmp/192824, PMID: 40407145

[34] Gomez K Santiago U Nelson TS Allen HN Calderon-Rivera A Hestehave S . A peptidomimetic modulator of the Ca_V_2.2 N-type calcium channel for chronic pain. Proc Natl Acad Sci USA. (2023) 120:e2305215120. doi: 10.1073/pnas.2305215120 37972067 PMC10666126

[35] Loya-Lopez SI Allen HN Duran P Calderon-Rivera A Gomez K Kumar U . Intranasal CRMP2-Ubc9 inhibitor regulates Na V 1.7 to alleviate trigeminal neuropathic pain. Pain. (2024) 165:573–88. doi: 10.1097/j.pain.0000000000003053, PMID: 37751532 PMC10922202

[36] Prado-E-Silva L de Oliveira Melchior M Mélo AM Stuginski-Barbosa J Mazzi-Chaves JF Díaz-Serrano KV . Higher levels of dispositional mindfulness are associated with more effective ecological momentary intervention outcomes in reducing the frequency of awake bruxism behaviours. J Oral Rehabil. (2025) 52:859–70. doi: 10.1111/joor.13943, PMID: 39861974

[37] Atilgan E Kurt H Algun ZC. Effect of yoga-based exercise program in female patients with myofacial pain of temporomandibular disorders. Clin Oral Investig. (2024) 28:642. doi: 10.1007/s00784-024-06045-y, PMID: 39547987

[38] Jin L Yao Y Fang Z Fan S Cai B Xu L . Long-term prognosis and influencing factors of Chinese adolescents with temporomandibular disorder after physical therapy. J Oral Rehabil. (2024) 51:2611–21. doi: 10.1111/joor.13865, PMID: 39305035

[39] Val M Manfredini D Guarda Nardini L. Is botulinum toxin the future of orofacial pain management? Evidence and perspectives. Dent Med Probl. (2025) 62:405–7. doi: 10.17219/dmp/200127 40553080

[40] Sielski M Chęcińska K Turosz N Chęciński M Sikora M. Single intra-articular administration of injectable platelet-rich fibrin (I-PRF) in alleviating temporomandibular joint pain: a pilot clinical trial. Dent Med Probl. (2025) 62:187–92. doi: 10.17219/dmp/188273, PMID: 40043084

[41] Schiffman E Ohrbach R Truelove E Look J Anderson G Goulet JP . Diagnostic criteria for temporomandibular disorders (DC/TMD) for clinical and research applications: recommendations of the international RDC/TMD consortium network^*^ and orofacial pain special interest group^†^. J Oral Facial Pain Headache. (2014) 28:6–27. doi: 10.11607/jop.1151 24482784 PMC4478082

[42] Speciali JG Dach F. Temporomandibular dysfunction and headache disorder. Headache. (2015) 55:72–83. doi: 10.1111/head.12515, PMID: 25644695

[43] Sommer I Lavigne G Ettlin DA. Review of self-reported instruments that measure sleep dysfunction in patients suffering from temporomandibular disorders and/or orofacial pain. Sleep Med. (2015) 16:27–38. doi: 10.1016/j.sleep.2014.07.023, PMID: 25547038

[44] Cioffi I Perrotta S Ammendola L Cimino R Vollaro S Paduano S . Social impairment of individuals suffering from different types of chronic orofacial pain. Prog Orthod. (2014) 15:27. doi: 10.1186/s40510-014-0027-z, PMID: 24935241 PMC4047491

[45] Nadendla LK Meduri V Paramkusam G Pachava KR. Evaluation of salivary cortisol and anxiety levels in myofascial pain dysfunction syndrome. Korean J Pain. (2014) 27:30–4. doi: 10.3344/kjp.2014.27.1.30, PMID: 24478898 PMC3903798

[46] Chebbi R Chaabani I Alaya TB Dhidah M. Elongated styloid process as a cause of facial pain. Joint Bone Spine. (2014) 81:368. doi: 10.1016/j.jbspin.2014.03.007, PMID: 24746811

[47] Al-Harthy M Ohrbach R Michelotti A List T. The effect of culture on pain sensitivity. J Oral Rehabil. (2016) 43:81–8. doi: 10.1111/joor.12346, PMID: 26371794

[48] Neubert JK Widmer CG Malphurs W Rossi HL Vierck CJ Jr Caudle RM. Use of a novel thermal operant behavioral assay for characterization of orofacial pain sensitivity. Pain. (2005) 116:386–95. doi: 10.1016/j.pain.2005.05.011, PMID: 15982812

[49] Lindholm P Lamusuo S Taiminen T Pesonen U Lahti A Virtanen A . Right secondary somatosensory cortex-a promising novel target for the treatment of drug-resistant neuropathic orofacial pain with repetitive transcranial magnetic stimulation. Pain. (2015) 156:1276–83. doi: 10.1097/j.pain.0000000000000175, PMID: 25830924

[50] Torisu T Tanaka M Murata H Wang K Arendt-Nielsen L De Laat A . Modulation of neck muscle activity induced by intra-oral stimulation in humans. Clin Neurophysiol. (2014) 125:1006–11. doi: 10.1016/j.clinph.2013.10.018, PMID: 24238991

[51] Forssell H Jääskeläinen S List T Svensson P Baad-Hansen L. An update on pathophysiological mechanisms related to idiopathic oro-facial pain conditions with implications for management. J Oral Rehabil. (2015) 42:300–22. doi: 10.1111/joor.12256, PMID: 25483941

[52] Bakker NA Van Dijk JM Immenga S Wagemakers M Metzemaekers JD. Repeat microvascular decompression for recurrent idiopathic trigeminal neuralgia. J Neurosurg. (2014) 121:936–9. doi: 10.3171/2014.7.JNS132667, PMID: 25084464

[53] McDonough P McKenna JP McCreary C Downer EJ. Neuropathic orofacial pain: cannabinoids as a therapeutic avenue. Int J Biochem Cell Biol. (2014) 55:72–8. doi: 10.1016/j.biocel.2014.08.007, PMID: 25150831

[54] Deschaumes C Devoize L Sudrat Y Baudet-Pommel M Dualé C Dallel R. The relationship between resting arterial blood pressure and oral postsurgical pain. Clin Oral Investig. (2015) 19:1299–305. doi: 10.1007/s00784-014-1356-5, PMID: 25399874

[55] Cooper MS. Role of endocrine dysfunction in frequently unexplained disorders. Eur J Pain. (2014) 18:299–300. doi: 10.1002/j.1532-2149.2013.00429.x, PMID: 25728516

[56] Baad-Hansen L Pigg M Yang G List T Svensson P Drangsholt M. Reliability of intra-oral quantitative sensory testing (QST) in patients with atypical odontalgia and healthy controls—a multicentre study. J Oral Rehabil. (2015) 42:127–35. doi: 10.1111/joor.12245, PMID: 25284726 PMC4293308

[57] Zhang L Ji M Sun Y Wang Q Jin M Wang S . VTA dopaminergic neurons involved in chronic spared nerve injury pain-induced depressive-like behavior. Brain Res Bull. (2025) 222:111261. doi: 10.1016/j.brainresbull.2025.111261, PMID: 39956400

[58] Sannajust S Imbert I Eaton V Henderson T Liaw L May M . Females have greater susceptibility to develop ongoing pain and central sensitization in a rat model of temporomandibular joint pain. Pain. (2019) 160:2036–49. doi: 10.1097/j.pain.0000000000001598, PMID: 31430262 PMC7092504

[59] Sangalli L Souza LC Letra A Shaddox L Ioannidou E. Sex as a biological variable in oral diseases: evidence and future prospects. J Dent Res. (2023) 102:1395–416. doi: 10.1177/00220345231197143, PMID: 37967405

[60] Santos SAAR Damasceno MBMV Sessle BJ Vieira-Neto AE de Oliveira Leite G Magalhães FEA . Sex differences in the orofacial antinociceptive effect of metformin and the role of transient receptor potential channels. Naunyn Schmiedeberg’s Arch Pharmacol. (2024) 398:3775–88. doi: 10.1007/s00210-024-03475-z 39356320

[61] Li X Ge SN Li Y Wang HT. Neurokinin-1 receptor-immunopositive neurons in the medullary dorsal horn provide collateral axons to both the thalamus and parabrachial nucleus in rats. Neurochem Res. (2017) 42:375–88. doi: 10.1007/s11064-016-2080-0, PMID: 28097463

[62] Wilcox SL Gustin SM Macey PM Peck CC Murray GM Henderson LA. Anatomical changes within the medullary dorsal horn in chronic temporomandibular disorder pain. NeuroImage. (2015) 117:258–66. doi: 10.1016/j.neuroimage.2015.05.014, PMID: 25979666

[63] Ahmed N Mustafa HM Catrina AI Alstergren P. Impact of temporomandibular joint pain in rheumatoid arthritis. Mediat Inflamm. (2013) 2013:597419. doi: 10.1155/2013/597419, PMID: 24363501 PMC3864075

[64] Kroese JM Volgenant CMC van Schaardenburg D van Boheemen L van Selms MKA Visscher CM . Oral health-related quality of life in patients with early rheumatoid arthritis is associated with periodontal inflammation and painful temporomandibular disorders: a cross-sectional study. Clin Oral Investig. (2022) 26:555–63. doi: 10.1007/s00784-021-04034-z, PMID: 34279701 PMC8791886

[65] Lawal FB John MT Oladayo AM Paulson DR Theis-Mahon N Ingleshwar A. Oral health impact among children: a systematic review update in 2024. J Evid Based Dent Pract. (2025) 25:102082. doi: 10.1016/j.jebdp.2024.102082, PMID: 39947784

[66] Fernando Oyarzo J Manriquez C Durham J. Cross cultural validation of oral health index profile for temporomandibular disorders in Spanish speaking population. J Oral Rehabil. (2025) 52:137–43. doi: 10.1111/joor.13881, PMID: 39482889

[67] Yekkalam N Sipilä K Novo M Reissmann D Hanisch M Oelerich O. Oral health-related quality of life among women with temporomandibular disorders and hypermobile Ehlers–Danlos syndrome or hypermobility spectrum disorder. J Am Dent Assoc. (2024) 155:945–53. doi: 10.1016/j.adaj.2024.08.013 39352367

[68] Yang G Jin J Wang K Baad-Hansen L Liu H Cao Y . Effect of lingual nerve block and localised somatosensory abnormalities in patients with burning mouth syndrome-a randomised crossover double-blind trial. J Oral Rehabil. (2024) 52:453–63. doi: 10.1111/joor.13877 39496499

[69] Xiaojie X Yiling C Honglei L Jiamei P Xiaoyong W Hao Y . Comparative analysis of myoelectric activity and mandibular movement in healthy and nonpainful articular temporomandibular disorder subjects. Clin Oral Investig. (2024) 28:605. doi: 10.1007/s00784-024-05957-z, PMID: 39428401

[70] Chattrattrai T Janal MN Lobbezoo F Raphael KG. The association between sleep bruxism and awake bruxism: polysomnographic and electromyographic recordings in women with and without myofascial pain. J Oral Rehabil. (2023) 50:822–9. doi: 10.1111/joor.13468, PMID: 37073471 PMC10524115

[71] de Oliveira-Souza ALS Gülker L Tavares LF Andrade AV Dennett L Fuentes J . The effectiveness of aerobic exercise compared to other types of treatment on pain and disability in patients with orofacial pain: a systematic review. J Oral Rehabil. (2024) 51:2696–735. doi: 10.1111/joor.13823, PMID: 39313927

[72] Romeo A Incorvati C Vanti C Turolla A Marinelli F Defila L . Physical therapy in addition to occlusal splint in myogenic temporomandibular disorders: a randomised controlled trial. J Oral Rehabil. (2024) 51:1566–78. doi: 10.1111/joor.13729, PMID: 38757854

[73] Hsieh YL Yang CC Yang NP. Ultra-low frequency transcutaneous electrical nerve stimulation on pain modulation in a rat model with myogenous temporomandibular dysfunction. Int J Mol Sci. (2021) 22:9906. doi: 10.3390/ijms22189906, PMID: 34576074 PMC8465049

[74] Elkholy MAE Abd-Elsayed A Raslan AM. Supraorbital nerve stimulation for facial pain. Curr Pain Headache Rep. (2023) 27:157–63. doi: 10.1007/s11916-023-01113-6, PMID: 37129764 PMC10198823

[75] Woda A Pionchon P. A unified concept of idiopathic orofacial pain: clinical features. J Orofac Pain. (1999) 13:172–84. 10823031

[76] Ono K Harano N Nagahata S Seta Y Tsujisawa T Inenaga K . Behavioral characteristics and c-Fos expression in the medullary dorsal horn in a rat model for orofacial cancer pain. Eur J Pain. (2009) 13:373–9. doi: 10.1016/j.ejpain.2008.05.004, PMID: 18599327

[77] Woda A Pionchon P. A unified concept of idiopathic orofacial pain: pathophysiologic features. J Orofac Pain. (2000) 14:196–212. 11203755

[78] Jang IS Nakamura M. Pregnenolone sulfate potentiates tetrodotoxin-resistant Na channels to increase the excitability of dural afferent neurons in rats. J Headache Pain. (2025) 26:42. doi: 10.1186/s10194-025-01968-7 40000932 PMC11863801

[79] Watanabe M Shrivastava RK Balchandani P. Advanced neuroimaging of the trigeminal nerve and the whole brain in trigeminal neuralgia: a systematic review. Pain. (2025) 166:282–310. doi: 10.1097/j.pain.0000000000003365, PMID: 39132931 PMC12782627

[80] Clemente-Napimoga JT Mendes V Trindade-da-Silva CA Carvalho G Paranhos ACGA Andrade E Silva F . Experimental traumatic occlusion drives immune changes in trigeminal ganglion. Int Immunopharmacol. (2023) 122:110674. doi: 10.1016/j.intimp.2023.110674, PMID: 37481846

[81] Da Antunes Da Cunha T Chaves TC Pereira Júnior FJ De Gonçalves DAG Alstergren P Biasotto-Gonzalez DA. Brazilian Portuguese version of the diagnostic criteria for temporomandibular disorders axis II: translation, cross-cultural adaptation and measurement properties. J Oral Rehabil. (2025) 52:712–21. doi: 10.1111/joor.13921, PMID: 39888088

[82] von Piekartz H Bleiss S Herzer S Hall T Ballenberger N. Does combining oro-facial manual therapy with bruxism neuroscience education affect pain and function in cases of awake bruxism? A pilot study. J Oral Rehabil. (2024) 51:1692–700. doi: 10.1111/joor.13740, PMID: 38894567

[83] Christidis N Al-Moraissi EA Al-Ak’hali MS Minarji N Zerfu B Grigoriadis A . Psychological treatments for temporomandibular disorder pain-a systematic review. J Oral Rehabil. (2024) 51:1320–36. doi: 10.1111/joor.13693, PMID: 38616535

[84] Müggenborg F de Castro Carletti EM Dennett L de Oliveira-Souza AIS Mohamad N Licht G . Effectiveness of manual trigger point therapy in patients with myofascial trigger points in the orofacial region-a systematic review. Life. (2023) 13:336. doi: 10.3390/life13020336, PMID: 36836693 PMC9965624

[85] Siewert-Gutowska M Pokrowiecki R Kamiński A Zawadzki P Stopa Z. State of the art in temporomandibular joint arthrocentesis-a systematic review. J Clin Med. (2023) 12:4439. doi: 10.3390/jcm12134439, PMID: 37445474 PMC10342956

[86] Busse JW Casassus R Carrasco-Labra A Durham J Mock D Zakrzewska JM . Management of chronic pain associated with temporomandibular disorders: a clinical practice guideline. BMJ. (2023) 383:e076227. doi: 10.1136/bmj-2023-076227, PMID: 38101929

[87] Dipalma G Inchingolo AD Pezzolla C Sardano R Trilli I Di Venere D . The association between temporomandibular disorders and tinnitus: evidence and therapeutic perspectives from a systematic review. J Clin Med. (2025) 14:881. doi: 10.3390/jcm14030881, PMID: 39941552 PMC11818186

[88] Häggman-Henrikson B Lövgren A Wu WY Peck C Westergren H List T. Prevalence of temporomandibular disorder symptoms after whiplash trauma-a systematic review and meta-analysis. Eur J Pain. (2025) 29:e4792. doi: 10.1002/ejp.4792, PMID: 39921489 PMC11806439

[89] Qataya PO Zaki AM Amin F Swedan A Elkafrawy H. Piano level laser therapy versus epidermal growth factor injection for painful myogenic temporomandibular disorder (a randomized clinical trial). Clin Oral Investig. (2025) 29:118. doi: 10.1007/s00784-025-06189-5, PMID: 39912963 PMC11802707

[90] Lövgren A Vallin S Häggman-Henrikson B Kapos FP Peck CC Visscher CM . Women are worse off in developing and recovering from temporomandibular disorder symptoms. Sci Rep. (2025) 15:4732. doi: 10.1038/s41598-025-86502-0, PMID: 39922904 PMC11807177

[91] Kutschke A Bechmann B Häggman-Henrikson B Wänman A Durham J Lövgren A. Exploring the patients’ perspective on digital tools for psychosocial assessment in dentistry. J Oral Rehabil. (2025) 52:495–505. doi: 10.1111/joor.13909, PMID: 39871666 PMC11934849

[92] Schreiber V Kunz M Achterberg W van der Steen JT Lobbezoo F Langner B . Development and validation of a short version (PAIC6) of the pain assessment in impaired cognition scale. Eur J Pain. (2025) 29:e4795. doi: 10.1002/ejp.4795, PMID: 39923123 PMC11807239

[93] Macedo de Sousa B López-Valverde N López-Valverde A Neves D Santos M Rueda JAB. Effect of dry needling, ischemic compression and cross-taping of the masseter in patients with orofacial myofascial pain: a randomized comparative study. Front Oral Health. (2025) 5:1524496. doi: 10.3389/froh.2024.1524496, PMID: 39839663 PMC11747114

[94] Mu W Li S Lu Q Wang J Tao X. The immediate pain relief of low-level laser therapy for burning mouth syndrome: a retrospective study of 94 cases. Front Oral Health. (2024) 5:1458329. doi: 10.3389/froh.2024.1458329, PMID: 39744416 PMC11688308

[95] Mansoori M Rocha Exposto C Hammer Bech B Frodi Olsen S Ahrendt Bjerregaard A Baad-Hansen L. Is poor dietary quality in adolescence a risk factor for painful temporomandibular disorders and headaches in young adulthood? A prospective study in the Danish National Birth Cohort. Headache. (2025) 65:731–44. doi: 10.1111/head.14899, PMID: 39905724

[96] Stundys D Kučinskaitė A Gervickaitė S Grigaitienė J Tutkuvienė J Jančorienė L. Exploring the role of symptom diversity in facial basal cell carcinoma: key insights into preoperative quality of life and disease progression. Cancers. (2025) 17:138. doi: 10.3390/cancers17010138, PMID: 39796765 PMC11720226

[97] Walumbe J Denneny D. Reframing pain care: an equity lens on psychosocial and behavioural interventions. Curr Opin Psychol. (2025) 62:102001. doi: 10.1016/j.copsyc.2025.102001, PMID: 39921948

[98] Manfredini D Bender SS Häggman-Henrikson B Durham J Greene CS. Temporomandibular disorders: a new list of key points to summarize the standard of care. Jpn Dent Sci Rev. (2025) 61:1–2. doi: 10.1016/j.jdsr.2024.12.001, PMID: 39816712 PMC11729766

[99] Kao H Wiryasaputra R Liao YY Tsan YT Chu WM Chen YH . The potential for high-priority care based on pain through facial expression detection with patients experiencing chest pain. Diagnostics. (2024) 15:17. doi: 10.3390/diagnostics15010017, PMID: 39795545 PMC11720015

[100] Dagli N Haque M Kumar S. A bibliometric analysis of clinical trials on salivary biomarkers for mental health (2003–2024). Cureus. (2024) 16:e64635. doi: 10.7759/cureus.64635, PMID: 39021745 PMC11253590

[101] Guo XJ Wu P Jia X Dong YM Zhao CM Chen NN . Mapping the structure of depression biomarker research: a bibliometric analysis. Front Psychiatry. (2022) 13:943996. doi: 10.3389/fpsyt.2022.943996, PMID: 36186850 PMC9523516

[102] Wang X Li X Dong T Yu W Jia Z Hou Y . Global biomarker trends in triple-negative breast cancer research: a bibliometric analysis. Int J Surg. (2024) 110:7962–83. doi: 10.1097/JS9.0000000000001799, PMID: 38857504 PMC11634138

[103] Huang K Lidbury BA Thomas N Gooley PR Armstrong CW. Machine learning and multi-omics in precision medicine for ME/CFS. J Transl Med. (2025) 23:68. doi: 10.1186/s12967-024-05915-z, PMID: 39810236 PMC11731168

[104] Dybing KM McAllister TW Wu YC McDonald BC Broglio SP Mihalik JP . Association of Alzheimer’s disease polygenic risk score with concussion severity and recovery metrics. Sports Med. (2025) 55:1487–503. doi: 10.1007/s40279-024-02150-w, PMID: 39821585 PMC12152024

[105] Ozsari S Güzel MS Yılmaz D Kamburoğlu K. A comprehensive review of artificial intelligence based algorithms regarding temporomandibular joint related diseases. Diagnostics. (2023) 13:2700. doi: 10.3390/diagnostics13162700, PMID: 37627959 PMC10453523

[106] Bodenreider O. The Unified Medical Language System (UMLS): integrating biomedical terminology. Nucleic Acids Res. (2004) 32:D267–70. doi: 10.1093/nar/gkh061, PMID: 14681409 PMC308795

[107] Campillos-Llanos L Valverde-Mateos A Capllonch-Carrión A Moreno-Sandoval A. A clinical trials corpus annotated with UMLS entities to enhance the access to evidence-based medicine. BMC Med Inform Decis Mak. (2021) 21:69. doi: 10.1186/s12911-021-01395-z, PMID: 33618727 PMC7898014

[108] Jiang Y Yu J Fu L Liu Y Li S Ding Y . Advances and frontiers in pulmonary fibrosis and lung cancer research (2000–2024): a bibliometric analysis. Front Med. (2025) 12:1596228. doi: 10.3389/fmed.2025.1596228, PMID: 40625354 PMC12229879

[109] Marshall IJ Nye B Kuiper J Noel-Storr A Marshall R Maclean R . Trialstreamer: a living, automatically updated database of clinical trial reports. J Am Med Inform Assoc. (2020) 27:1903–12. doi: 10.1093/jamia/ocaa163, PMID: 32940710 PMC7727361

[110] Schiffman E Ohrbach R Truelove E Look J Anderson G Goulet JP . International RDC/TMD Consortium Network, International association for Dental Research; Orofacial Pain Special Interest Group, International Association for the Study of Pain. Diagnostic Criteria for Temporomandibular Disorders (DC/TMD) for Clinical and Research Applications: recommendations of the International RDC/TMD Consortium Network* and Orofacial Pain Special Interest Group†. J Oral Facial Pain Headache.. (2014) 28:6–27. doi: 10.11607/jop.1151, PMID: 24482784 PMC4478082

